# Machine learning for extracellular vesicles enables diagnostic and therapeutic nanobiotechnology

**DOI:** 10.1186/s12951-025-03952-4

**Published:** 2026-01-17

**Authors:** Ashutosh Tiwari, Widodo, Dyah Ika Krisnawati, Kai-Yi Tzou, Tsung-Rong Kuo

**Affiliations:** 1 International Ph.D. Program in Biomedical Engineering, College of Biomedical Engineering, Taipei Medical University, Taipei, 11031 Taiwan; 2 Institut Teknologi AI-Mahrusiyah, Kediri, East Java, 64112 Indonesia; 3 Department of Nursing, Faculty of Nursing and Midwifery, Universitas Nahdlatul Ulama Surabaya, Surabaya, 60237 Indonesia; 4 Center for Continuing Care Research (C3R), Universitas Nahdlatul Ulama Surabaya, 60237, Surabaya East Java, Indonesia; 5 Department of Urology, Shuang Ho Hospital, Taipei Medical University, New Taipei City, 23561 Taiwan; 6 Department of Urology, School of Medicine, College of Medicine, Taipei Medical University, Taipei, 11031 Taiwan; 7 Taipei Medical University Research Center of Urology and Kidney, Taipei Medical University, Taipei, 11031 Taiwan; 8 Graduate Institute of Nanomedicine and Medical Engineering, College of Biomedical Engineering, Taipei Medical University, Taipei, 11031 Taiwan; 9 Precision Medicine and Translational Cancer Research Center, Taipei Medical University Hospital, Taipei, 11031 Taiwan

**Keywords:** Extracellular vesicles (EVs), Smart nanotherapeutics, Machine learning in nanomedicine, Bioinspired nanomaterials, AI driven materials design, Targeted drug delivery platforms

## Abstract

Extracellular vesicles (EVs) are emerging as naturally bioactive nanomaterials with intrinsic biocompatibility and targeting potential. Recent integration of machine learning (ML) into EV research has accelerated advances in molecular profiling, structure–function prediction, and rational design of vesicle-based therapeutics. Yet, the inherent complexity and heterogeneity of EV populations pose major analytical challenges. Concurrently, machine learning is revolutionizing biomedical science by uncovering patterns in high dimensional, multimodal datasets. In EV research, ML has enabled major advances across automated imaging, multi omics integration, disease classification, therapeutic engineering, and standardization. This review presents a comprehensive synthesis of ML-enabled EV studies, organized by data modality (imaging, omics, cytometry), algorithmic paradigm (CNNs, random forests, autoencoders, GNNs), and translational application (diagnosis, prognosis, drug delivery, manufacturing QC). Unlike prior reviews that have typically considered EV biology and AI methods in relative isolation, we introduce a unified three-axis taxonomy that explicitly links EV data modalities, machine learning architectures, and clinical use-cases, thereby providing a structured map of the field. We discuss key technical barriers including data sparsity, batch variability, and model explainability and spotlight frontier developments such as federated learning, self-supervised models, and real-time EV analytics. At the nexus of computational intelligence and nanomedicine, ML-enhanced EV platforms are rapidly progressing from fragmented innovations to clinically actionable systems. This review offers a roadmap for advancing AI-integrated EV technologies in cancer precision medicine.

**Graphical abstract:**

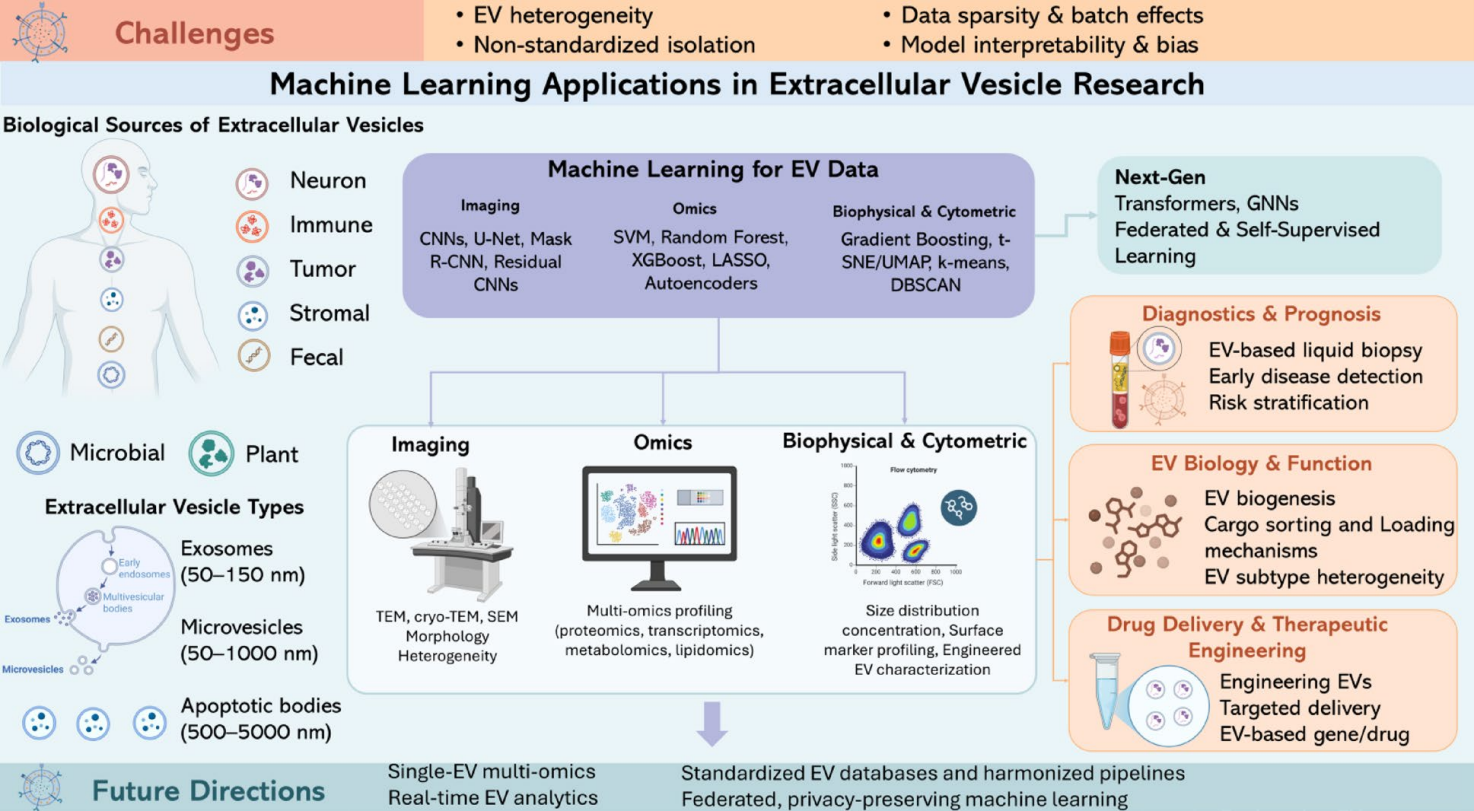

## Introduction

### Definition and importance of extracellular vesicles (EVs)

EVs are a heterogeneous collection of membrane enclosed nanoparticles (NPs) that are released by cells and have only recently been recognized [1] as key mediators of intercellular communication within physiological and pathological processes [2]. Vesicles are generally divided into exosomes, microvesicles (MVs) [3], and apoptotic bodies, with the type determined by the source, size, molecular composition, and mechanism of release [4].

Exosomes are the smallest of the EV categories, with diameters ranging 30–150 nm [5]. They are derived from the endosomal compartment through the inward budding of multivesicular bodies (MVBs) [6], which then fuse with a plasma membrane to expel exosomes into the extracellular environment. MVs, by contrast, are relatively larger, ranging 50–1000 nm, and are produced through the direct outward budding from a plasma membrane [7]. Apoptotic bodies, ranging 500–5000 nm in size, are shed during the final stages of programmed cell death and contain fragmented DNA and other cellular organelles [8]. Although these size ranges have traditionally been used as a basis for classification, growing recognition of the profound overlap between EV sizes, where differences are more often operational rather than strictly absolute, has created a need for more sophisticated systems of classification [9].

EVs consist of a heterogeneous and dynamic set of biomolecules, such as proteins, lipids, nucleic acids ranging from messenger (m)RNAs, micro (mi)RNAs, long non coding (lnc)RNAs, to sometimes DNA and metabolites, which often reflect the physiological or pathological state of their parent cells [10, 11]. EV molecular cargos are not random but are produced by an active cellular process that is modulated by the cellular context, types of environmental stress, and certain regulatory processes [12, 13], such as the endosomal sorting complex required for transport (ESCRT) and tetraspanin enriched microdomains [14, 15].

EVs play important roles in maintaining tissue homeostasis and orchestrating complex cellular responses [16]. They are involved in numerous processes, such as angiogenesis, immune modulation, tissue repair, and neuronal signaling, thus placing themselves as important players in systemic intercellular communication [17, 18]. For instance, in the immune system, EVs from dendritic cells have the capacity to present antigens to T cells, thus acting as antigen presenting vehicles [19]. In oncology, tumor derived EVs (TDEs) were implicated in promoting metastasis, modifying the tumor microenvironment (TME), and promoting immune evasion [20–22].

In addition, EVs exhibit high stability in biological fluids like blood, urine, saliva, and cerebrospinal fluid, mainly due to their ability to resist enzymatic degradation, which is a result of their shielding lipid bilayer [22]. This stability, along with their molecular cargos, renders EVs highly significant as minimally invasive biomarkers, especially in the context of liquid biopsies for early disease diagnoses [2].

Over the last decade, the biomedical relevance of EVs has moved from a topic of interest to one of substantial clinical relevance. The International Society for Extracellular Vesicles (ISEV) has highlighted the need for terminological and methodological standardization, leading to the Minimum Information for Studies of EVs (MISEV) guidelines that define the criteria for characterizing EVs [23]. These efforts demonstrate the growing consensus that EVs are not cell remnants but powerful biological entities and useful diagnostic tools, with enormous potential for breakthroughs in therapeutic strategies.

### Machine learning (ML) in biomedical research

ML, a key part of artificial intelligence (AI), refers to computational algorithms that can recognize patterns and make predictions or decisions from data without being explicitly programmed [24]. Essentially, ML uses statistical methods to “learn” from data by iteratively improving objective functions to maximize performances by working with structured and unstructured datasets over time [25]. ML models are divided into three distinct categories: supervised learning, where associations between inputs and outputs are discovered using pre-defined outputs [26]; unsupervised learning, which identifies hidden patterns in the absence of labeled outcomes [27]; and reinforcement learning, where agents learn optimal actions through trial and error in changing environments [28].

ML has radically reoriented the field of biomedicine by enabling the extraction of meaningful conclusions from complicated, high dimensional, heterogeneous, and often noisy datasets that characterize modern day biological information [29]. Traditional statistical methods are often unable to handle such scenarios, particularly when the number of features like genes, proteins, and pixels in imaging data considerably exceeds the number of observations, i.e., patients or samples [30]. In contrast, ML shows great capabilities to handle such datasets by applying methods like dimensionality reduction, regularization, and advanced optimization methods [31]. The principal driver for adopting ML in biomedical studies is the staggering deluge of multi omics data, ranging from genomics, transcriptomics, proteomics, and metabolomics [32, 33]. Advanced high throughput technologies like next generation sequencing (NGS) and mass spectrometry have facilitated this reality. These novel techniques produce data that are dominated by complexity and quantity, creating the need to use computational methods capable of finding patterns, extracting features, and predicting outcomes [34]. ML programs have been fundamental in applications including cancer subtype annotation, mutation impact predictions, and drug response modeling, among numerous others [35, 36]. Additionally, the area of medical imaging encompassing radiology, histopathology, and cellular microscopy has undergone progressive shifts through the application of ML, particularly with the advent of deep learning (DL) methods. Convolutional neural networks (CNNs) are now able to match or surpass the performance of human experts in tasks like tumor detection on radiographs, histological feature segmentation, and single cell image phenotyping [37, 38]. This has accelerated diagnostic workflows, reduced variability between different observers, and enabled the creation of automated decision support systems in clinical environments [39].

ML is also used in electronic health records (EHRs) to help combine longitudinal patient information to allow for real time risk stratification, patient classification, and the creation of individualized treatment plans [40]. In addition, natural language processing (NLP), a subdivision of ML, has allowed the collection of pertinent information from unstructured clinical reports, thus enhancing the accuracy of phenotyping and disease trend monitoring [41].

ML is key to investigating poorly explored biological spaces, such as the case of extracellular vesicles (EVs), in which traditional approaches often face limitations because of the inherent heterogeneity of the field and the lack of standardized data frameworks [42]. By integrating imaging, molecular, and biophysical information relevant to EVs, ML algorithms can discern patterns that escape human observation, thereby improving our understanding of EV biology and its translational potential.

Despite its potential to revolutionize the field, the application of ML in biomedicine requires rigorous validation, interpretability, and strict ethical supervision. Issues like bias in training data, a lack of transparency in model predictions (the so called “black box” problem), and challenges in generalizability across various populations are key areas for examination [43]. However, with the advancement of computing power, coupled with advancements of data availability and algorithms, ML is set to be an integral part of biomedical research and clinical innovation.

### Intersection of ML and EV research

The convergence of ML and EV research is an emerging area of inquiry with profound implications for basic biological investigations as well as the clinical utility of scientific discovery. The rationale for applying ML to the study of EVs is based on the inherent variability and heterogeneity of these objects, the heterogeneity of related data types (e.g., omics, imaging, and biophysical attributes), and the urgent need for robust, scalable, data driven analytical frameworks capable of providing biologically and clinically relevant insights [44].

EVs act as biomarkers of the physiological or pathological status of their parent cells, due to their dynamic molecular contents. However, their small size, heterogeneity of biogenesis, and shared physical characteristics with other subcellular structures like lipoproteins and protein aggregates pose major analytical challenges [45]. In addition, the molecular contents of EVs, including proteins, nucleic acids, lipids, and metabolites, are not only cell type specific but are also determined by the external environment, requiring thorough, high dimensional analyses beyond the capacity of conventional statistical methods [46]. Standard analytical models often fail to cope with the nonlinear, multiscale, and multimodal nature of EV datasets, regardless of their origin from mass spectrometry based proteomics, NGS, or transmission electron microscopy (TEM) [47].

The field of ML provides a powerful solution to challenges inherent in this domain. Through the use of algorithms tailored for feature extraction, dimensionality reduction, and predictive modeling, ML enables the discovery of biomarker signatures, classification of EV subtypes, prediction of disease states, and modeling of EV mediated intercellular communication with high accuracy [48]. In research using imaging methods for EVs, CNNs have shown excellent performance in the automatic detection, segmentation, and phenotypic characterization of EVs in complex backgrounds, thus reducing the need for time consuming manual annotation commonly required for such tasks [49, 50]. In molecular studies, ensemble learning methods, including random forests (RFs) and extreme gradient boosting (XGBoost), as well as support vector machines (SVMs), have proven to be efficient for disease classification based on profiles obtained from EV associated miRNAs, protein expression matrices, and spectral signatures.

What sets this review apart is threefold. First, we aim to categorize and critically evaluate the different types of data produced within EV research, including imaging, omics, cytometric, and spectroscopic methods, highlighting their unique analytical requirements. Second, we provide a systematic overview of ML methods applied across these data types, including their theoretical underpinnings, performance metrics, and application specific considerations. Third, we discuss the clinical and translational potential of ML augmented EV research, including non-invasive diagnostics, drug delivery optimization, and disease monitoring, as well as methodological challenges like EV heterogeneity, data sparsity, standardization gaps, and model interpretability. Beyond this structured taxonomy, we explicitly foreground three emerging yet underrepresented paradigms in EV research: graph neural networks for EV-related interaction and communication networks, federated and privacy-preserving learning for multi-institutional datasets, and real-time or streaming analytics for on-chip and point-of-care EV monitoring, thereby outlining a forward-looking agenda that extends beyond prior EV–AI reviews.

To conclude, this review projects into the future landscape of ML EV convergence, identifying emerging trends such as real time EV analytics, single EV profiling, multi-omics data fusion, and AI driven therapeutic EV engineering. By presenting a comprehensive and forward-looking analysis, we aim to equip researchers, clinicians, and data scientists with the conceptual and methodological tools necessary to advance the frontiers of EV research through ML.

## Types of data in EV research

EV studies now cover many types of data, from very small-scale imaging and large-scale omics to single-EV cytometry and functional or clinical results. Instead of giving a full technical review of each platform [51], this part shortly describes the main EV data types, their usual measurement methods, and the kind of biological info they hold. These summaries give the needed background for the next part, where we look at how different machine learning methods use these data to deal with specific analytical problems.

### Imaging data

Imaging is an integral component of research involving EVs, as it provides visual confirmation of their presence, and their complex morphological features, size ranges, and structural complexity. While biochemical and molecular methods recognize the composition of EVs, imaging techniques provide vital spatial and morphological information that is essential for their classification and quality evaluation [52]. Due to the nanometric size of EVs, most of which measure < 200 nm, the use of electron microscopy and imaging equipment with nanoscale resolution is unavoidable for their detailed visualization [53, 54]. Imaging modalities most commonly used in the study of EVs include transmission electron microscopy (TEM), scanning electron microscopy (SEM), and cryogenic (cryo) TEM, among which each modality has its own advantages and limitations [55]. TEM has been considered the gold standard for imaging EVs due to its exceptional resolution power, which can go below 1 nm, and its ability to depict complex structural features of single vesicles [56]. In conventional negative staining approaches, EVs are largely seen as cup shaped or spherical structures, a morphology that was previously considered diagnostic but is now recognized as an artefact of dehydration and preparation procedures [57]. TEM is often used for verifying the presence of vesicles, assessing the purity of EV preparations, and discriminating EVs from contaminants like protein aggregates and lipoproteins [58]. However, the reliance of the technique on heavy metal staining and its vulnerability to artifacts generated by sample preparation have prompted the development of more advanced techniques.

**Fig. 1:**
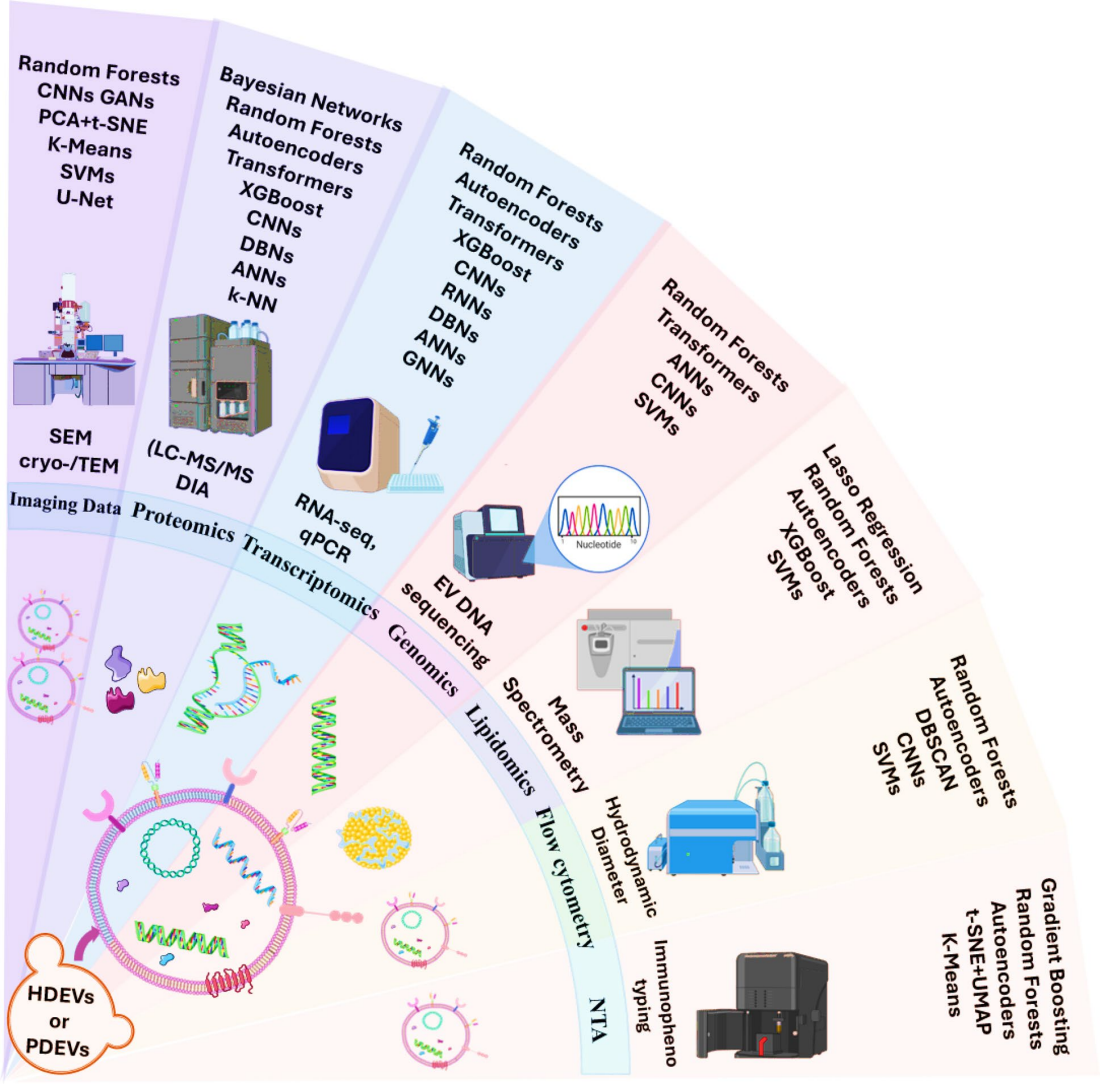
Summary of ML applications to EV data modalities. The figure illustrates main ML techniques to the main EV data types like imaging (SEM, cryo TEM), proteomics, transcriptomics, genomics, lipidomics, flow cytometry, and NTA. HDEVs and PDEVs stand for human and plant derived EVs. ML models ranging from classical algorithms (e.g., SVM, Random Forests) to deep learning (e.g., CNNs, Transformers) are coupled with domain specific tasks like classification, segmentation, and feature extraction

Cryo TEM, an extension of conventional TEM, overcomes many of its limitations by embedding vesicles in vitreous ice without staining, thus maintaining their native morphology and ultrastructure [59]. Cryo TEM has revealed that EVs often display characteristics of membrane multilayering, intraluminal vesicles, and irregular morphologies––features not previously observable with standard TEM protocols. Such architectural heterogeneity underscores the biologic diversity of EV populations and suggests that particular biophysical subtypes can correlate with various cellular origins or biologic functions [60]. That said, in spite of its power, cryo TEM requires special instrumentation, cryogenic conditions, and specialized interpretation, and thus its availability is limited. SEM, although not commonly used, provides complementary information by imaging the surface topography and enabling analysis of vesicle aggregates on different substrates and cellular surfaces [61]. SEM has been particularly useful for surface characterization of vesicle secretion surfaces, evaluating EVs and synthetic scaffold interactions in biomaterials science, and confirming vesicle morphology in correlative studies [62]. In addition to qualitative visualization, imaging data are increasingly being used for quantitative and computational analyses, including through the application of ML supported image processing pipelines. Deep learning (DL) based approaches, and in particular convolutional neural networks (CNNs) [63], have shown high accuracy in the segmentation and classification of EVs from TEM datasets, substantially reducing manual annotation requirements and improving reproducibility [64]. Novel approaches, including fast radon based U Net (FRU Net), have enabled the rapid and precise detection of small EVs in complex or noisy microscopic images with a tolerance to shape variations and improved throughput [49]. Similarly, Mask R CNN [65]based models (e.g., ScanEV) have been trained on carefully annotated EV image datasets to automate a range of processes, including instance segmentation, morphometric analyses, and population level heterogeneity assessments [50]. Imaging is an important tool in phenotypic classification of EVs through measurement of their size, contour aberrations, and intravesicular structure, thus identifying differences in biogenic routes (e.g., endosomal sorting complex required for transport (ESCRT) dependent vs. ESCRT independent routes) or in cellular stress levels [66]. As the field moves toward characterizing individual EVs, high resolution imaging will remain indispensable, not just for confirming morphological findings, but also as groundwork for developing multimodal ML models that link imaging with biochemical and molecular data [67]. In summary, electron microscopy provides an indispensable visual framework for EV research, enabling the characterization of vesicle morphology, purity, and heterogeneity. With the integration of ML based image analysis tools, imaging data are now transitioning from static visualizations to dynamic, quantifiable, and scalable datasets, thereby catalyzing deeper insights into EV biology and classification.

### Omics data

The complex molecular structure of EVs is best explained by the use of omics technologies, which provide in depth, high throughput information regarding EV contents [68, 69]. Such approaches enable quantitative and qualitative analyses of proteins, nucleic acids, lipids, and metabolites, each with unique information regarding the function, origin, and clinical relevance of EVs [70]. The information obtained with omics not only is critical for understanding the biological role of EVs but also for identifying biomarkers and therapeutic targets relevant to a wide range of diseases [71].

#### Proteomics

EV proteomics applies techniques like liquid chromatography tandem mass spectrometry (LC MS/MS) and data independent acquisition (DIA) to detect and quantify the entire range of proteins encapsulated inside or displayed on EV surfaces. The range covers tetraspanins (cluster of differentiation 9 (CD9), CD63, and CD81), heat shock proteins, integrins, and cytoskeletal elements, to mention but a few [72]. Proteomes of EVs are type and condition specific, hence reflecting the physiological or pathological cell conditions from which the EVs were derived [73].

In oncology, proteomic profiling of EVs has enabled the discovery of tumor derived protein signatures with diagnostic and prognostic potential. For instance, plasma factor 4 (PF4) and alpha 1 antichymotrypsin (AACT) proteins identified via a random forest (RF) based ML model of serum EVs demonstrated high discriminatory power in early colorectal cancer (CRC) detection, outperforming classical tumor markers like carcino embryonic antigen (CEA) and carbohydrate antigen 19 9 (CA19 9) [74]. Similarly, proteomic analyses of EVs in neurodegenerative diseases (NGDs) have identified cargos such as annexin A5 (ANXA5) and nerve growth factor inducible (VGF) as potential biomarkers for Alzheimer’s disease (AD), capturing disease relevant brain activity from peripheral samples [75, 76].

#### Transcriptomics

Transcriptomic profiling of EVs focuses primarily on small RNA species, including miRNAs, small nucleolar (sno)RNAs, and occasionally long non coding (lnc)RNAs and messenger (m)RNAs [77, 78]. Techniques such as RNA Seq, quantitative polymerase chain reaction (qPCR) arrays, and NanoString are used to analyze EV RNA cargos, which are selectively enriched and protected within vesicles [79, 80]. Among these, miRNAs have garnered particular attention due to their regulatory roles in gene expression and stability in biofluids. EV-derived miRNA panels have been implicated in a variety of cancers; for example, elevated levels of miR 21, miR 1246, and miR 423 5p in EVs were respectively associated with breast, prostate, and pancreatic cancers [81–83]. In cardiovascular and inflammatory disorders, distinct miRNA cargo profiles were correlated with disease severity and response to therapy, positioning EVs as liquid biopsy tools for precision medicine [84, 85].

#### Genomics

Less well explored than EV RNA, EV DNA, including double stranded genomic DNA and mitochondrial (mt)DNA, has become a newly discovered and valuable cargo. Its presence was documented in cancer derived EVs and can represent somatic mutations of tumor origin [86, 87]. EV DNA Seq of the complete genome or targeted regions offers a non-invasive method for detecting mutational patterns in diseases like lung or colon cancer, possibly complementing or even surpassing traditional cell free DNA studies [88–90]. However, the field of EV genomics remains in its infancy, with open questions regarding DNA packaging processes, biological relevance, and purity of isolation.

#### Lipidomics

The lipid composition of EVs, although often overlooked, plays crucial roles in membrane fluidity, vesicle formation, and target cell interactions. EV membranes are enriched in sphingomyelin, cholesterol, phosphatidylserine, and ceramides, which not only confer structural integrity but also influence cellular uptake and biodistribution [91, 92]. Lipidomic profiling using mass spectrometry (MS) has been used to distinguish EV subtypes, characterize disease associated lipid alterations, and even predict EV drug carrying potential [92, 93]. In metabolic disorders and NGDs, changes in the lipid profiles of EVs were linked to underlying pathologic processes. For instance, EVs isolated from patients with Parkinson’s disease exhibit significant changes in their lipid saturation levels, suggesting that lipidomics may serve as a functional biomarker for monitoring disease progression and neuronal stress [94, 95].

New advances in microfluidics, plasmonics, and signal amplification technologies have facilitated the single-cell level lipidomic analysis of EVs [96] from very minute biofluid volumes, thus avoiding the limitations of bulk analyses. Such platforms are crucial for the discovery of cell-type-specific vesicular signatures and determining the heterogeneity of EV-mediated signaling. New advances in microfluidics, surface nano-engineering, and MS-based detection have facilitated highly sensitive EV lipidomic analyses from very small biofluid volumes, thereby mitigating the averaging effects of bulk measurements. In parallel, intact-particle lipidomics has enabled detailed characterization of exosomal membranes in cancer. For example, Lobasso et al. [97] applied matrix-assisted laser desorption/ionization time-of-flight MS (MALDI-TOF/MS) to profile melanoma cells and their secreted exosomes, demonstrating that exosomes are enriched in sphingomyelin, lysophosphatidylcholine, phosphatidic acid, and bis(monoacylglycero)phosphate, and identifying bis(monoacylglycero)phosphate as a specific phospholipid marker of melanoma-derived exosomes. Moreover, their analysis revealed systematic differences in fatty acyl chain length and saturation between poorly and highly metastatic melanoma cells, highlighting the potential of EV lipid signatures as biomarkers of metastatic propensity and tumor behavior. Together, omics technologies facilitate multidimensional EV cargo characterization with functional insights into vesicle biogenesis, target specificity, and systemic signaling. In conjunction with ML models, omics data inform powerful classification systems and predictive algorithms for early diagnosis, disease stratification, and monitoring therapeutic responses [98, 99]. However, variability in EV isolation techniques, platform sensitivity, and sample preparation protocols presents a challenge to data reproducibility and cross study comparison issues currently being resolved through international standardization initiatives such as Minimal Information for Studies of EVs 2023 (MISEV2023) [23, 100].

### Other data types: biophysical and cytometric profiling of EVs

Along with imaging and molecular omics, biophysical measurements derived using a nanoparticle tracking analysis (NTA) and flow cytometry form part of critical quantitative parameters required for the characterization, standardization, and quality assurance of EVs [101]. These techniques are widely used to analyze size distributions, particle concentrations, and surface marker expressions, among several other characteristics, in an effort to distinguish subpopulations in heterogeneous EV preparations [102, 103]. While such types of information (Fig. 1) are sometimes referred to as “ancillary” to omics or imaging pipelines, they are in fact pivotal for guaranteeing compliance and reproducibility, and for driving preclinical translational research, especially in areas of biomarker discovery and drug development [104].

#### Nanoparticle (NP) tracking analysis (NTA)

NTA is a light scattering technique that follows the Brownian motion of individual NPs visualized using a microscope, enabling their hydrodynamic diameter and concentration to be calculated via the Stokes Einstein equation [105]. NTAs have been widely adopted in EV studies, enabling high throughput measurements of particle size distributions across approximately 30 to 1000 nm and making it suitable for the analysis of the full EV range (including exosomes and microvesicles) in purified and raw biological samples [101, 106]. Even with its ubiquitous use, NTA is faced with limitations in specificity, as it is incapable of distinguishing EVs from non-vesicular particles like lipoproteins, protein aggregates, or exomeres [107]. To allow for phenotype specific detection, e.g., CD63⁺ EVs, scientists have utilized fluorescent labeling methods (fluorescent NTA); however, ongoing hurdles related to dye stability, background noise, and spectral overlap remain major challenges [108]. Additionally, variations in equipment and the user dependent nature of thresholding indicate the need for standardized methods and better calibration strategies, areas where ML might play a major role by automating gate calculations and improving signal resolution.

#### Flow cytometry

Flow cytometry, particularly its high sensitivity or nanoscale forms, has emerged as a useful tool for analyzing single EVs, allowing for surface protein marker identification, membrane integrity assessments, and relative measurements of different EV subtypes [109]. Compared to NTA, which provides limited phenotypic data, flow cytometry offers the ability for multiparametric immunophenotyping when used in conjunction with fluorescently labeled antibodies against recognized EV markers (e.g., CD9, CD81, and CD63) or targeted cellular antigens [110]. Traditional flow cytometric instruments were generally not sensitive enough to detect particles smaller than about 300 nm, and thus smaller populations of EVs were underrepresented [111]. However, developments like flow cytometers dedicated to NPs (e.g., Apogee A60 Micro and NanoFCM NanoAnalyzer) and improvements in scatter detection methods have enabled quantitative measurements down to 50 nm, essentially lowering the detection limit for EVs [112].

Flow cytometry is a critical component of quality control (QC) in EV isolation workflows, including quantifying remaining cellular debris or aggregates, and is growing in significance in clinical assay development [113]. One example is the Extracellular Vesicle Machine Learning Analysis Platform (EVMAP), which combines microscale flow cytometry and ML algorithms to discriminate EVs derived from prostate cancer from those of healthy donors using a multivariate panel of fluorescence features [114]. Such approaches underscore the synergy of high dimensional cytometric data with ML in disease classification, patient stratification, and risk assessment.

#### Toward standardization and integration

As EV-based diagnostics and therapeutics move closer to clinical translation, the need for robust, reproducible, and quantitative assays has become paramount. Both NTA and flow cytometry contribute critical QC parameters, such as particle to protein ratios, event counts per volume, and expression heterogeneity, which are increasingly mandated by regulatory and publication standards (e.g., MISEV2018 and MISEV2023 guidelines) [23, 115]. Additionally, such information offers a strong basis for including ML methods, particularly in areas of classification, clustering, and anomaly detection. For instance, unsupervised learning methods (t distributed stochastic neighbor embedding (t SNE), uniform manifold approximation and projection (UMAP), and k means) are used to visualize and classify populations of EVs based on their multidimensional marker expressions, while supervised models (XGBoost and RFs) predict disease classifications or treatment outcomes based on features obtained from cytometry [103, 116]. In brief, NTA and flow cytometry serve as complementary and orthogonal techniques that take EV characterization beyond morphology and molecular composition. When properly deployed, especially coupled with ML approaches, they provide the needed quantitative accuracy and phenotypic distinction necessary to standardize EV research, increase reproducibility, and advance clinical translation.

## ML techniques applied to EV data

This section builds on the EV data types presented in Sect. "Types of Data in EV Research" and looks at how machine learning is adapted for specific analysis needs. For each main data type, imaging (Sect. "Imaging Data"), omics (Sect. "Omics Data"), single-EV and cytometric profiling (Sect. "Other Data Types: Biophysical and Cytometric Profiling of EVs"), and functional/clinical readouts (Sect. "Toward Standardization and Integration"), we discuss common ML models, the problems they solve (like detection, clustering, classification, feature selection), and important progress made possible by these models.

### Techniques for imaging data

The application and use of deep learning (DL) techniques have changed dramatically, especially with a very strong focus on convolutional neural networks (CNNs) and their different variants (Table 1). This has dramatically altered and revolutionized the way imaging data are being analyzed particularly in the field of EV research [117, 118]. The emergence of advanced imaging technologies, including TEM and cryo TEM, has led to the production of extremely high resolution but also very complex visual datasets. In this regard, DL is a priceless computational toolset and offers the necessary infrastructure that researchers require to efficiently decode quantitative information and also complex high resolution details from EV images [119]. This decoding is achieved at an incredible level of speed, accuracy, and reproducibility, which is far better than what is achievable by manual analysis or by traditional computer vision methods that were previously used [120]. Several machine learning models, including CNNs, appear across different sections of this review. This is intentional, as each appearance reflects a unique application domain ranging from image segmentation in EV microscopy [121] to omics-based feature selection and cytometry classification. Rather than consolidating these models into a general section, we preserve their modality-specific context to highlight technological variation and practical implementation nuances.

#### CNNs in EV segmentation and classification

CNNs, characterized by their ability to capture spatial hierarchies of features through layered convolutional filters, are particularly well suited to tasks such as EV segmentation, shape detection, and classification in microscopic images [50]. In EV studies, CNNs have enabled the development of end to end pipelines that automate the labor intensive process of identifying EVs amid background noise, cellular debris, or other nano structural challenges that routinely limit throughput and reproducibility in manual workflows [122]. A foundational initial contribution involved using U Net architectures for EV segmentation from TEM images. Such neural networks, specifically designed for biomedical image segmentation tasks, exhibit a capability for successful learning from limited data while retaining spatial resolution through skip connections [49]. Building on this idea, the fast Radon based U Net (FRU Net) incorporated Radon transforms in order to enhance edge detection and structural representation in low contrast EV images, thus enabling accurate segmentation of various shapes and intensity gradients [123]. Further innovation has come through residual CNNs, which improve performance by mitigating the vanishing gradient problem in deeper networks. These architectures enable more nuanced feature extraction, particularly for subtle morphological traits such as membrane asymmetry or intraluminal vesicle features that may correlate with the EV subtype or biogenesis pathway [124].

**Table 1:** AI model summary for EV data analysis

AI Model	Applicable Domains	Primary Use Cases	Data Modalities	Data Types	Data Formats	Key References
Artificial neural networks	Genomics, proteomics, transcriptomics	Gene interaction modeling; predictive modeling; variant impact modeling	Bulk RNA Seq, epigenomics, shotgun proteomics	Gene expression counts, genomic variants, sequences, peptide/protein abundances	FASTQ, BAM, CSV, HDF5, VCF, BED, mzML, mzXML,	[125, 126]
Autoencoders	Flow cytometry, imaging, lipidomics, NTA, proteomics, transcriptomics	Denoising cytometry signal; dimensionality reduction; image denoising; latent feature encoding; noise filtering; spectral denoising	FACS, MS, NTA, shotgun lipidomics, TEM, EM, scRNA Seq	Gene expression counts, lipid intensities/profiles, microscopic images, peptide/protein abundances, signal intensity, events, size, concentration distributions	CSV, Excel, JSON, FASTQ, BAM, HDF5, FCS, TIFF, PNG, JPEG, mzML, TXT, mzML, mzXML,	[2, 127–130]
Bayesian networks	Proteomics	Probabilistic inference	PPI networks	Peptide/protein abundances	mzML, mzXML, CSV	[131]
Convolutional Neural Networks (CNNs)	Flow cytometry, genomics, proteomics, transcriptomics	Epigenetic analysis; pattern detection in EV populations; spatial detection; spatial proteomics patterning	CRISPR screening, imaging flow cytometry, imaging mass cytometry, spatial transcriptomics	Gene expression counts, genomic variants, sequences, peptide/protein abundances, signal intensity, events	FASTQ, BAM, HDF5, FCS, CSV, VCF, FASTQ, BED, mzML, mzXML,	[132, 133]
CNNs	Imaging	Feature extraction in EV microscopy	TEM, SEM	Microscopic images	TIFF, PNG, JPEG	[134, 135]
DBSCAN	Flow cytometry	Anomaly detection	CyTOF	Signal intensity, events	FCS, CSV	[136, 137]
Deep Belief Networks	Proteomics, transcriptomics	Deep feature hierarchy; hierarchical learning	Deep RNA Seq, MS data	Gene expression counts, peptide/protein abundances	FASTQ, BAM, CSV, HDF5, mzML, mzXML,	[138, 139]
Gradient Boosting	NTA	Concentration estimation/biomarker	NTA	Size, concentration distributions	CSV, Excel, JSON	[140, 141]
K means	Imaging, NTA	Subpopulation clustering; unsupervised clustering, proteomic meta analysis	NTA, TEM	Microscopic images, size, concentration distributions	CSV, Excel, JSON, TIFF, PNG, JPEG	[142, 143]
Lasso Regression	Lipidomics	Sparse regression modeling	NMR	Lipid intensities/profiles	mzML, CSV, TXT	[144, 145]
PCA + t SNE	Metabolome	Dimensionality reduction	RNA Seq	Gene expression counts, peptide/protein abundances	FASTQ, BAM, CSV, HDF5, mzML, mzXML, CSV	[146, 147]
Recurrent Neural Networks	Transcriptomics	Temporal modeling	Time series RNA Seq	Gene expression counts	FASTQ, BAM, CSV, HDF5	[148, 149]
Random Forests	Flow cytometry, genomics, imaging, lipidomics, NTA, proteomics, transcriptomics	Classification; feature importance; feature ranking; gating strategy optimization; gene classification; lipid profile classification; size distribution modeling	Flow cytometry, fluorescence, LC MS/MS, MS lipidomics, NTA, RNA Seq, whole genome Seq	Gene expression counts, genomic variants, sequences, lipid intensities/profiles, microscopic images, peptide/protein abundances, signal intensity, events, size, concentration distributions	CSV, Excel, JSON, FASTQ, BAM, HDF5, FCS, TIFF, PNG, JPEG, VCF, BED, mzML,, TXT, mzXML,	[150–153]
Support Vector Machines	Flow cytometry, genomics, imaging, lipidomics, proteomics, transcriptomics	Biomarker detection; classification; EV phenotype classification; feature classification; risk prediction	2D GE, high dimensional flow, microarrays, nanostring, TEM, SEM, targeted LC MS	Gene expression counts, genomic variants, sequences, lipid intensities/profiles, microscopic images, peptide/protein abundances, signal intensity, events	BAM, HDF5, FCS, CSV, TIFF, PNG, JPEG, VCF, BAM, BED, TXT, mzML, mzXML,	[154–156]
Transfer Learning	Imaging	Pretrained model adaptation	Any	Microscopic images	TIFF, PNG, JPEG	[157]
Transformers	Genomics, proteomics, transcriptomics	Contextual embedding; sequence interpretation; sequence modeling	Long read Seq, long read sequencing, proteogenomics	Gene expression counts, genomic variants, sequences, peptide/protein abundances	FASTQ, BAM, CSV, HDF5, VCF, BED, mzML, mzXML,	[158]
U Net	Imaging	Semantic segmentation	Cryo EM	Microscopic images	TIFF, PNG, JPEG	[159]
Extreme Gradient Boosting (XGBoost)	Lipidomics, proteomics, transcriptomics	High dimensional classification; lipidomics subtype prediction; subtype prediction	SWATH MS, untargeted lipidomics, mRNA arrays	Gene expression counts, lipid intensities/profiles, peptide/protein abundances	FASTQ, BAM, CSV, HDF5, TXT, mzML, mzXML,	
k Nearest Neighbor	Proteomics	Phenotype classification	Peptide fingerprinting	Peptide/protein abundances	mzML, mzXML, CSV	[160]
t SNE + UMAP	NTA	Dimensionality reduction for visualization	NTA	Size, concentration distributions	CSV, Excel, JSON	[161]

RNA Seq: RNA sequencing, scRNA Seq: single cell RNA sequencing, NTA: nanoparticle tracking analysis, FACS: fluorescence activated cell sorting, CyTOF: cytometry by time of flight, MS: mass spectrometry, TEM/SEM/EM/Cryo EM: Transmission/Scanning/Electron/Cryogenic Electron Microscopy, LC MS/MS: liquid chromatography tandem mass spectrometry, PPI: protein–protein interaction, 2D GE: two dimensional gel electrophoresis, NMR: nuclear magnetic resonance spectroscopy, FCS: flow cytometry standard file format, VCF: variant call format, BED: browser extensible data format, mzML/mzXML: mass spectrometry data formats, BAM/FASTQ: genomic sequence formats, CSV/HDF5/JSON/TXT/Excel: standard data file formats, CRISPR: clustered regularly interspaced short palindromic repeats, t SNE/UMAP/PCA: dimensionality reduction techniques, AI/ML/DL: artificial intelligence / machine learning / deep learning, CNN/RNN/SVM/XGBoost: convolutional/recurrent neural networks, support vector machine, eXtreme Gradient Boosting

#### Cryo TEM and heterogeneity analysis

While conventional TEM imaging often distorts EV morphologies due to fixation and staining artifacts, cryo TEM preserves EVs in a near native, hydrated state. However, the visual complexity and noise level in cryo TEM datasets present a significant analytical burden. To address this, CNNs and unsupervised DL models have been employed to classify structural subtypes of EVs and uncover hidden morphological heterogeneity that would otherwise be imperceptible through manual inspection [3]. Gómez de Mariscal et al. [49] developed a residual CNN combined with Radon transform preprocessing to segment small EVs in TEM images, achieving superior accuracy compared to traditional image analysis methods and enabling detailed morphological characterization at the single vesicle level. More recently, in a preprint, Kapoor et al. analyzed over 7500 individual EVs using cryo TEM imaging coupled with segmentation neural networks, revealing striking morphological heterogeneity across EV populations irrespective of their cellular origin or isolation technique [162]. This large-scale computational phenotyping supports the hypothesis that vesicle architecture is biologically encoded rather than a product of random variations. Together, those studies underscore the potential of DL based imaging analysis to refine current EV classification frameworks and enhance our understanding of vesicle biogenesis beyond conventional size based paradigms.

#### Toward Standardized, ML powered imaging workflows

More recently, models such as Mask R CNN have enabled instance level segmentation of EVs, allowing for precise morphometric analyses at the single vesicle level, including area, circularity, and aspect ratio. The ScanEV pipeline, built on Mask R CNN, demonstrated > 90% segmentation accuracy on annotated TEM datasets and achieved high generalization to unseen sample types, highlighting the robustness of DL in cross sample analyses [50]. In addition, the novel integration of cutting-edge DL techniques with specialized data augmentation methods optimized for specific applications, coupled with the successful utilization of transfer learning techniques from pre trained networks like ImageNet, and supplemented by the utilization of self-supervised learning techniques, has allowed researchers to achieve exceptionally high performance levels. This is especially relevant in situations where data are scarce, a common situation in studies involving EV images. These dramatic developments are driving a revolutionary paradigm shift away from conventional static descriptions, moving towards more computationally tractable approaches that enable high dimensional phenotyping of EVs. In this forward thinking paradigm, morphological features are utilized not just for the validation process but are also identified as features of biological significance [163]. These features can greatly enhance downstream ML applications, which include tasks such as classification, different types of clustering analyses, and modeling aimed at determining associations with diseases.

### Techniques for omics data

The omics characterization of EVs, encompassing proteomics, transcriptomics, genomics, and lipidomics, generates data that are of high dimensionality and high biological significance, but also high structural complexity. To extract meaningful patterns from this complex data, there is a need to apply both supervised and unsupervised ML techniques capable of dealing with feature redundancy, identifying relevant biomarkers, and facilitating disease classification toward clinical use [164–166].

#### Supervised learning methods

With supervised learning, models are constructed using labeled datasets where the outcome variable of interest, e.g., disease status or tissue origin, is predetermined. These models are central to the identification of biomarkers, EV-based diagnostics, and classification of disease subtypes [114]. Support vector machines (SVMs) are widely used because of their ability to handle high dimensional data and their skill at detecting subtle, nonlinear differences. In the field of EV transcriptomics, SVMs have successfully discriminated between miRNA profiles derived from malignant and nonmalignant plasma samples, frequently outperforming standard statistical classification methods [167]. Random forests (RFs), which comprise multiple decision trees, are prized for their interpretability and resistance to overfitting. In the investigation of serum EV proteomes for the purpose of early CRC detection, RF models were employed, with the illustration of platelet factor 4 (PF4) and alpha 1 antichymotrypsin (AACT) proteins as markers for early-stage colorectal cancer. The RF model had an area under the curve (AUC) of 0.96 in test sets, which highlighted its effectiveness in combining several markers [74]. Extreme gradient boosting (XGBoost) is an improvement over RFs by its use of boosting techniques that successively correct model errors. A recent study using EV flow cytometric data found that XGBoost based models exhibited considerably better sensitivity and specificity in prostate cancer detection compared to single marker models and traditional prostate specific antigen (PSA) tests [114, 168]. Logistic regression (LR), although simple in nature, remains relevant because of its interpretability and proficiency in merging EV features with clinical factors. Often, it acts as the final layer in ensemble architectures that are used for risk estimation, such as multimodal EV diagnostic pipelines [169]. Least Absolute Shrinkage and Selection Operator (LASSO regressions are particularly useful for variable selection in omics datasets that are subject to multicollinearity. Through the addition of a penalty on the size of coefficients, LASSO reduces the number of variables incorporated into the model, making it easier to determine the most informative variables within noisy EV data [170].

#### Unsupervised learning approaches

Unsupervised learning is used to explore the intrinsic structure of EV data without predefined labels. This is particularly useful in generating hypotheses, discovering EV subpopulation, and visualizing sample clustering [116]. Principal component analysis (PCA) is a widely used statistical method for dimensionality reduction while also preserving the variance in the dataset. It is often used as the first step in many omics analytical workflows, which are used to enable the visualization of sample clustering based on factors like disease status, tissue of origin of the samples, or the technique employed for EV isolation [171]. Clustering algorithms, including different techniques like k means clustering and hierarchical clustering, are utilized to effectively classify and stratify samples of EVs or different molecular features into several distinct subgroups to predict cardiovascular risks [172]. These subgroups are identified on the basis of inherent similarities within the data. For instance, a group of researchers who performed hierarchical clustering of miRNA expression profiles of EVs isolated from patients with breast cancer were able to successfully identify unique clusters. These clusters were significantly associated with particular clinical subtypes of breast cancer. The subtypes identified included luminal A, human epidermal growth factor receptor 2 (HER2) enriched, and triple negative breast cancer (TNBC), as was identified in earlier research reports [173].

In recent integrative studies, unsupervised clustering followed by supervised classification was used as a two-step approach to define and validate biologically meaningful EV signatures. For example, EV-derived proteomes were first clustered into latent groups, which were then used as features in downstream SVM and RF models to classify tumor types and stages with high fidelity [174].

#### Applications and example studies

The fusion of omics data and ML has enabled reliable classification of disease states, predictions of treatment responses, and early detection of malignancies using minimally invasive samples such as blood, saliva, or urine.

- In one large scale study, serum derived EVs were profiled using four dimensional data independent acquisition (4D DIA) mass spectrometry, and an ML pipeline involving an RF and LR identified a panel of proteins capable of distinguishing benign from malignant colorectal lesions with an AUC of > 92% [175].
- Similarly, EV-derived miRNA panels demonstrated high predictive value in pancreatic ductal adenocarcinoma, where ensemble learning models like XGBoost achieved an AUC value of 0.95 and accuracy of 92% in blinded test sets [176].
- Emerging studies have applied multi omics integration combining EV proteomics and transcriptomics to improve model robustness and capture pathway level perturbations in diseases such as AD and multiple sclerosis, areas where traditional diagnostic methods are invasive or limited in specificity [177, 178].

### Techniques for other data types

Imaging and omics fields directly impact the analysis of EVs; however, the quantitative information generated by biophysical characterization methods, specifically NTAs and flow cytometry, is very valuable. The effectiveness of these methods is further strengthened by the use of ML and pattern recognition techniques. These methods allow for strong and scalable assessments of EV sizes, concentration levels, surface marker expressions, and sample purity, thus streamlining QC procedures and developing predictive models for diagnostics [101, 179]. Table 2 provides a comparative overview of representative AI and machine-learning studies using extracellular vesicles in oncology, summarizing the application context, core algorithms, EV sources and data modalities, and the main diagnostic or mechanistic performance achieved.

#### ML with NTAs

NTAs yield time resolved light scattering data, which can then be translated into particle size distributions and concentration profiles. Although traditionally used as a QC tool to determine the yield of EV isolation and heterogeneity in size, NTA data can also serve as predictive input for ML algorithms, especially if combined with metadata incorporating variables of disease state, isolation methods, or donor phenotypes [180, 181].

Supervised ML algorithms, such as SVMs and LR, have also recently been used to distinguish between EV samples obtained from healthy donors and from individuals with disease features, solely on the basis of parameters derived from an NTA, such as mean diameter, mode size, and concentration slopes [182–184]. In one particular case, an NTA based classifier was able to accurately identify patients with CRC compared to non-cancer controls at a rate of over 85%, suggesting that subtle changes in EV population dynamics have diagnostic value [185].

In addition, unsupervised learning methods such as PCA and clustering have been applied for batch effect detection and assessing inter sample heterogeneity, allowing standardization of EV preparations. These methods are especially important for producing clinical grade EV products, where regulatory standards require consistency and reproducibility in both size and purity [186].

#### ML for flow cytometry based EV analyses

Advancements in nanoscale flow cytometry have unlocked the ability to analyze EVs at the single particle level, enabling multiparametric assessment of surface markers, particle sizes, and fluorescence intensities. The resulting datasets are inherently high dimensional and well suited for ML driven pattern recognition [187, 188]. An exemplary application of ML assisted extracellular vesicle cytometry is the Extracellular Vesicle Machine Learning Analysis Platform (EVMAP), an integration of high sensitivity flow cytometry with ML that identifies disease specific EV signatures [189]. In this context, over 7 supervised ML models, including RFs, XGBoost, SVMs, and k nearest neighbors (k NNs), were tested to differentiate PSA samples obtained from prostate cancer patients and normal donors [190].

Therefore, unsupervised ML methods like t distributed stochastic neighbor embedding (t SNE) and uniform manifold approximation and projection (UMAP) have been used to visualize the heterogeneity in EV subpopulations derived from multicolor flow cytometric data. These methods effectively reduce the dimensionality of fluorescence data without losing local and global structures, allowing scientists to detect EV phenotypes that were not detectable before but could be of significant clinical or biological importance [191, 192].

#### Integrative applications and future potential

Detecting patterns in such data goes beyond static analysis. Several studies have explored longitudinal modeling using flow cytometry derived EV data to track disease progression or treatment responses using recurrent neural networks (RNNs) or time series regression approaches [193]. This method of dynamic modeling allows the creation of EV-based companion diagnostics and improves real time clinical monitoring. Nonetheless, combining NTA and flow cytometry features into multimodal ML pipelines has shown promise for enhancing diagnostic accuracy. For example, integrating EV size distributions from NTA with fluorescence marker intensities from flow cytometry can improve the specificity of disease classifiers while accounting for sample heterogeneity and platform limitations [194, 195]. In brief, ML based approaches are increasingly becoming indispensable in recognizing meaningful patterns in NTA and flow cytometric datasets [196]. These approaches are transforming traditionally secondary QC practices into biologically relevant and diagnostically valid sources of information, thus ensuring the achievement of clinical grade precision, reproducibility, and scalability for EV-based applications [188, 197].

**Table 2:** Artificial intelligence and machine-learning applications using extracellular vesicles for cancer detection, classification, and mechanistic analysis

Application Area	ML Algorithm/Method	EV Source/Sample Type	Key Findings/Performance	Data Type/Technology	References
Early diagnosis and stage discrimination of colorectal cancer (HC vs. polyp vs. early-stage CRC) using plasma EV proteomics	Feature selection with Random Forest (Gini importance) to select 10 protein markers; classification with Support Vector Machine (SVM) (compared against Naive Bayes, KNN, Random Forest, Classification Tree, BP neural network)	Plasma-derived EVs from healthy controls, patients with adenomatous polyps, and early-stage (stage I–II) CRC; discovery cohort *n* = 30 (HC = 11, polyp = 8, CRC = 11); independent single-blind validation cohort *n* = 28 (HC = 10, polyp = 8, CRC = 10)	DSPE-functionalized beads enabled rapid (∼10 min) EV isolation with broad, reproducible proteome coverage; SP3 + DIA-MS quantified 826 EV proteins and identified dysregulated signatures across disease stages. A 10-protein EV panel trained with SVM achieved 100% accuracy in internal validation and 89.3% accuracy in an independent single-blind cohort, with ROC AUCs up to 1.0 for key pairwise group separations, supporting EV-based proteomics as a fast, accurate, and cost-effective CRC screening strategy.	Plasma EV proteomics using DSPE-functionalized bead enrichment, SP3 sample preparation, and DIA-MS (DIA-NN-based analysis)	[198]
Serum EV-based diagnosis of colorectal cancer (CRC), including early-stage CRC and differentiation from benign colorectal disease (BCD) and inflammatory conditions	ML pipeline combining OPLS-DA for feature screening, RF, SVM, KNN, decision tree, and logistic regression; final EV-related Random Forest (RF) diagnostic model based on PF4, AACT, CEA, and CA19-9 (with prior RF + Lasso selection of PF4 and AACT from 4D-DIA proteomics)	Serum-derived EVs from multiple cohorts: discovery set (*n* = 37; 25 CRC, 12 HC), train set (*n* = 338; 96 HC, 47 BCD, 195 CRC), test set (*n* = 328; 112 HC, 55 BCD, 161 CRC), and external validation set (*n* = 246; 60 HC, 42 enteritis, 46 hepatitis B, 98 CRC)	4D-DIA EV proteomics identified 854 proteins and 12 candidate markers; PF4 and AACT emerged as key EV-derived biomarkers. RF models based on PF4/AACT outperformed traditional CEA and CA19-9. The final EV-related RF model (PF4 + AACT + CEA + CA19-9) achieved AUC = 0.960, PRAUC = 0.979, CE = 0.08 in the train set, and AUC = 0.963, PRAUC = 0.975, accuracy = 0.883 in the test set, with robust performance in an external cohort (accuracy = 0.810) and strong discrimination of early-stage (I–II) CRC from HC and CRC from BCD/inflammatory disease. PF4 and AACT levels correlated with stage and decreased after treatment, supporting both diagnostic and monitoring utility.	Serum EV proteomics via 4D-DIA (diaPASEF on timsTOF Pro, Spectronaut analysis) from ultracentrifugation- and SEC-isolated EVs, with ELISA validation of PF4 and AACT; integrated with TCGA RNA-seq, scRNA-seq, and GSEA-based functional analyses	[74]
Raman spectroscopy–based diagnosis of hepatocellular carcinoma (HCC) using exosome fingerprints, integrated into an interactive AI agent	Deep learning on Raman spectra using a Feature Fusion Transformer (FFT) with patch-based 1D self-attention and downsampling for exosome classification; wrapped in an LLM-based RAG agent (ChatExosome) that interprets user intent and calls spectroscopy-analysis plugins	Exosomes derived from HCC cell lines and clinical samples; Raman spectra from cell-derived exosomes and 165 clinical exosome samples	FFT model achieved 95.8% accuracy on cell-derived exosomes and 94.1% accuracy on 165 clinical exosome samples, enabling high-performance HCC vs. non-HCC discrimination from Raman fingerprints. Integrated into ChatExosome, an LLM-centered AI agent that uses retrieval-augmented generation to provide interactive, interpretable spectroscopic analysis, invoke appropriate processing tools, and support clinicians in HCC diagnosis by combining exosome Raman classification with up-to-date exosome knowledge.	Raman fingerprinting of exosomes; deep-learning FFT architecture for 1D spectral data; LLM-based interactive agent with retrieval-augmented generation (RAG) and plugin/tool invocation for Raman data processing	[199]
Binary classification of lung cancer vs. normal lung cell–derived EVs	Supervised ML using PCA + SVM with Bayesian hyperparameter optimization (linear kernel; optimized box constraint and kernel scale)	Exosomes from human non-small cell lung cancer cells (A549) and normal lung epithelial cells (BEAS-2B), isolated by ultracentrifugation	After MPLS baseline correction and DFT smoothing of SERS spectra, PCA showed good clustering; optimized SVM achieved CVloss 3.7% (5-fold cross-validation) and 98.7% test accuracy, misclassifying only 1/80 test spectra; AUC for both classes ≈ 0.9993, demonstrating highly accurate EV-based discrimination between A549 and BEAS-2B using small sample sizes (135 spectra per class).	Label-free liquid-phase SERS on AuNP (44.06 nm) substrates in capillaries; Raman spectra (633 nm laser) of EV–AuNP mixtures; MPLS baseline removal + DFT smoothing; PCA for feature exploration	[200]
Multi-class classification of EVs from five different cell lines	Classical ML using SVM (multi-class) and deep learning using 1D CNN	EVs from A549 (NSCLC), BEAS-2B (lung epithelial), HEK (embryonic kidney), HeLa (cervical cancer), HepG2 (liver cancer), all isolated by ultracentrifugation	SVM model: CVloss 0%, test accuracy 99.5% with recalls of 100%, 98.0%, 100%, 100%, and 100% for A549, BEAS-2B, HEK, HeLa, and HepG2, respectively; ROCs with AUC ≈ 1.0 for all classes. CNN model: using convolution–ReLU–pooling + fully connected + softmax, achieved test accuracy 100%, CVloss 0.4%, and AUC = 1.0 for all five EV classes, indicating near-perfect spectral discrimination across multiple tumor and normal cell–derived EVs.	Label-free SERS spectra of EVs on optimized AuNP substrates; MPLS + DFT preprocessing; PCA used to identify discriminative bands (e.g., 1349, 1544, 2818, 2851, 2913 cm⁻¹) followed by SVM or CNN classification	[200]
Classification of mixed-ratio lung cancer/normal EV samples (A549 + BEAS-2B mixtures)	SVM with Bayesian optimization and CNN for deep learning	Mixed exosome samples composed of A549 and BEAS-2B EVs in four ratios (99:1, 90:10, 75:25, 50:50); 135 spectra per mixture type (total 600 spectra)	SVM model on mixed samples: CVloss 8.3%, test accuracy 93.9%, with recalls of 93.2%, 95.7%, 93.3%, and 93.3% across the four mixture ratios and AUC values ~ 0.995 for each class. CNN model: CVloss 17.8%, test accuracy 85.6%, with recalls 86.3%, 72.5%, 86.0%, and 97.4% and AUC ≈ 0.91–0.98. Despite increased complexity vs. pure EV classes, ML models maintained high accuracy, supporting use in more realistic heterogeneous samples.	Label-free SERS on liquid EV–AuNP mixtures, preprocessed by MPLS + DFT; multi-class classification of mixture ratios using optimized SVM and CNN architectures	[200]
Plasma-derived EV classification in lung cancer vs. healthy mice	SVM and CNN models trained on SERS spectra	Plasma-derived EVs from lung cancer mouse model vs. healthy mice; 8 tumor and 8 healthy mice, each sample split into 5 replicates, 5 spectra/replicate (total 400 spectra)	SVM model: CVloss 2.5%, test accuracy 97.5%, recall 98.1% (lung cancer) and 97.0% (healthy), precision 96.4% and 98.5%, AUC ≈ 0.9879. CNN model: CVloss 5.0%, test accuracy 95.8%, recall 94.2% and 98.0%, precision 98.5% and 92.6%, AUC ≈ 0.9977. Demonstrates that plasma EVs in a murine lung cancer model can be classified with accuracy similar to cell-derived EVs despite complex blood environment.	Label-free liquid SERS of mouse plasma-derived EVs on AuNP substrates; MPLS + DFT preprocessing; PCA for exploration followed by SVM or CNN; evaluation via confusion matrices and ROC curves	[200]
Clinical plasma EV classification for early-stage lung cancer diagnosis	SVM and CNN applied to human plasma-derived EV SERS spectra	Plasma-derived exosomes from early-stage (stage I–II) lung cancer patients (*n* = 10) and healthy participants (*n* = 8); each sample divided into 10 replicates with 15 spectra/replicate, yielding 1500 (cancer) and 1200 (healthy) spectra (total 2700)	SVM model (Bayesian-optimized): CVloss 7.7%, test accuracy 91.5%; recall 95.4% (lung cancer) and 87.0% (healthy), precision 89.4% and 94.2%, AUC ≈ 0.9714. CNN model: CVloss 8.3%, test accuracy 95.4%; recall 97.6% and 92.8%, precision 94.4% and 96.8%, AUC ≈ 0.9916. Only 11/450 lung-cancer test spectra misclassified by CNN; results indicate strong potential for early lung cancer detection from clinical blood samples.	Label-free SERS on optimized 44.06 nm AuNP substrates with capillary-based liquid sampling (no drying); MPLS baseline removal + DFT smoothing; model interpretability via SHAP and PDP revealed key bands (e.g., 2851, 2913 cm⁻¹) linked to increased protein and metabolic signatures in cancer EVs	[200]
Global EV biochemistry interpretation from SERS + ML models	Model-agnostic explainability on SVM (and supportive PCA) using SHAP and Partial Dependence Plots (PDP)	Same human plasma-derived EV dataset (lung cancer vs. healthy) used above; SERS features across full spectral range	SHAP analysis ranked key Raman bands (≈ 2851, 1300, 1606, 1020, 2913, 1650, 1555–1587, 1349 cm⁻¹) as most influential for lung cancer classification; PDPs showed that increasing intensity at bands like 2913 cm⁻¹ (C–H stretching of lipids/proteins) increases predicted lung-cancer probability. Combined with band assignments, results suggest higher collagen and adenine levels and altered lipid/protein composition in cancer EVs vs. normal, providing biochemical insight beyond pure classification metrics.	Label-free SERS spectra; post-hoc model interpretation using SHAP and PDP on trained SVM classifier, integrated with prior peak assignment knowledge of lipid/protein bands	[200]
Pan-cancer *presence* detection (6 early-stage cancers vs. healthy)	1D CNN with Multiple Instance Learning (MIL) for binary classification (cancer vs. healthy). Signals from each sample (100 SERS spectra) treated as “instances”; sample label (0 = HC, 1 = cancer) used with MIL; sigmoid output averaged per sample as diagnostic score. Training with ~ 23k spectra (4,943 HC, 18,108 cancer) from 233 samples; 20% of training data used for validation; hyperparameters tuned by manual + random search.	Plasma exosomes isolated by size-exclusion chromatography (SEC) from: 210 healthy controls and 543 cancer patients (lung, breast, colorectal, liver, pancreatic, stomach). Retrospective, multi-center Korean cohort, pathologically confirmed, early-stage enriched (TNM 0–II, BCLC 0/A for liver).	On 520 independent test samples never seen during training, cancer presence classifier achieved AUC = 0.970 (95% CI 0.957–0.982), with sensitivity 89.4% and specificity 96.3% at optimal cutoff; overall accuracy 91.5%, precision 98.2%. Sensitivity at 99% specificity remained 72.5%, indicating robust performance under very stringent false-positive constraints. Per-cancer AUCs for presence detection in the test set: lung 0.936, breast 0.984, colorectal 0.972, liver 0.978, pancreatic 0.992, stomach 0.999. The CNN-MIL outperformed dummy and SVM baselines and remained stable across sex and cancer types.	Label-free SERS of plasma exosomes deposited on AuNP-aggregated array chips (100 nm AuNP, APTES-functionalized glass, centrifugation-assembled 2.5 mm spots). 10 measurement spots/chip; 100 spectra (10 × 10 grid) per sample, 785 nm excitation, 1 s integration, 50× objective. Preprocessing: denoising, baseline correction, spike removal; anomaly rejection if 860 cm⁻¹ band below threshold. SEC-isolated exosomes characterized by WB (CD9, CD63, CD81, TSG101), cryo-TEM, NTA (100–150 nm, 10⁹–10¹⁰ particles/mL).	[201]
Tissue-of-origin (TOO) classification across 6 cancers	Ensemble of CNN classifiers (one-vs-rest) for TOO discrimination, built on top of the cancer-positive spectra identified by the presence classifier. For each tumor type (lung, breast, colorectal, liver, pancreatic, stomach), a separate CNN model outputs a class-specific score; ROC evaluated one-vs-rest. Early-stage subgroup analysis done using TNM 0–II (or BCLC 0/A for liver).	Same plasma-exosome cohort as above; test subset for TOO: 278 early-stage cancer patients across six tumor types (lung, breast, colon, liver, pancreas, stomach) who were classified as cancer-positive by step-1 model.	For all test cancers, TOO models achieved a mean AUC ≈ 0.925 across one-vs-rest tasks with average sensitivity 87.4% and specificity 88.3%. In early-stage (0–II / BCLC 0–A) patients, TOO models retained high performance with mean AUC = 0.945. Per-type one-vs-rest AUCs for tissue classification (Table 2): lung 0.980, breast 0.876, colorectal 0.931, liver 0.907, pancreatic 0.913, stomach 0.941, with sensitivities generally ~ 0.71–0.98 and specificities ~ 0.75–0.95 depending on cancer type. CNN-based TOO classifiers outperformed dummy and SVM baselines, avoiding strong class biases observed with SVM for some cancers.	Same SEC-isolated plasma exosome SERS data and AuNP array platform as above. Input: sets of 100 SERS spectra per sample. Architecture: serial 1D convolutional layers + fully connected layers + softmax for multi-class prediction (one-vs-rest training). Structural ablation revealed strong dependence on the first convolutional layer (basic feature extraction of Raman spectra); performance degraded if first-layer filter size exceeded ~ 10, while later layers saturated at filter size ~ 5.	[201]
Integrated Exosome-SERS-AI decision system (cancer presence + TOO)	Two-stage CNN + MLP pipeline: (1) CNN-MIL binary classifier for cancer presence (as above); (2) six CNN one-vs-rest TOO classifiers; (3) MLP meta-classifier that takes six TOO scores and outputs final tumor site for cancer-positive samples. Decision rules: samples below cancer score cutoff to non-cancer; samples above cutoff to TOO decided by MLP over per-cancer scores to handle overlapping high scores and reduce false negatives.	Same multi-center plasma-exosome cohort; 520 previously unseen test samples (combining HC and all six cancer types and stages) used purely for evaluation of the integrated decision pipeline.	On the full 520-sample test set, the final integrated system achieved sensitivity 90.2% at specificity 94.4%, while correctly predicting the tissue of origin for 72% of cancer-positive patients. Stage-stratified analysis: for advanced-stage cancers, sensitivity reached 97.5%; for early-stage cancers, the system still delivered sensitivity 88.1% and TOO accuracy 75.9%. This demonstrates that a single Exosome-SERS-AI test can both flag early cancer and localize its organ of origin with clinically useful accuracy.	Label-free SERS of plasma exosomes, SEC isolation, AuNP array chips, 100 spectra/sample, 785 nm excitation; fully automated stage control and acquisition software in Python/pyQT5. Total lab workflow per sample: ~20 min SEC isolation + 30 min drying + 10 min SERS acquisition; AI decision is essentially instantaneous with pre-trained models, enabling sub-1-hour pan-cancer triage from a single blood tube.	[201]
Exosome-level spectral signatures and model behavior (explainability / design choices)	CNN-MIL architecture analysis + ablation, plus basic spectral band interpretation (not SHAP-level but structural). Baselines: dummy classifier and SVM.	Same plasma exosome SERS dataset. Signals are unlabeled at individual-spectra level but carry sample-level labels aggregated via MIL.	CNN-MIL significantly outperformed dummy and SVM baselines for cancer presence and TOO tasks. Ablation study showed that removing early convolutional layers, or increasing first-layer filter size above ~ 10, led to performance drops, highlighting importance of local Raman pattern extraction. Performance stable for dropout ≤ 0.5 and largely insensitive to fully-connected layer size. Spectral analysis identified cancer-associated bands common across groups near 691, 826, 938, 961, 993, 1136–1152, 1245, 1527, and 1595 cm⁻¹ (mostly protein-related), plus differences at 638, 668, 707, 733, 978, 1001, 1049, 1123, 1162, 1358, 1378, 1394, 1432 cm⁻¹ (proteins/lipids), supporting biological plausibility of AI-derived discrimination.	SERS platform as above; difference spectra between HC and cancer used to identify shared cancer-associated Raman bands. Model development in Python (scikit-learn, TensorFlow 2.5, Keras); ROC, AUC, PRC computed with scikit-learn; stats with scipy + pingouin.	[201]
Identification of universal exosome protein markers across cancers and controls	No classifier here – large-scale integrative proteomics analysis of public datasets; PCA for heterogeneity; frequency-based marker selection	Cell line–derived exosomes: 285 samples (228 cancer, 57 control) from multiple studies; tissue-derived exosomes: 157 samples (101 cancers, 56 controls)	From 1124 overlapping proteins across cell-line exosome datasets, 18 highly abundant exosome markers identified in ≥ 90% of samples; pathway analysis showed enrichment in vesicle-mediated transport, exocytosis, endocytosis etc. Comparing cell-line and tissue-derived exosomes, five proteins present in ≥ 90% of all samples (cell line + tissue): CLTC, EZR, TLN1, CAP1, MSN to proposed as universal exosome markers. Classical markers (CD63, TSG101, CD81, HSP70, etc.) were much less consistently detected in tissue exosomes (< 60% in many cases).	1124 overlapping proteins across cell-line exosome studies; 31 overlapping proteins at ≥ 90% frequency in both cell-line and tissue exosomes; 5 universal markers: Clathrin heavy chain (CLTC), Ezrin (EZR), Talin-1 (TLN1), Adenylyl cyclase-associated protein 1 (CAP1), Moesin (MSN). Cancer-specific marker from cell-line exosomes: ATXN2L detected in cancer exosomes but in ≤ 10% of control exosomes.	[202]
Pan-cancer detection (cancer vs. control) using plasma/serum exosome proteomes	Random Forest classifier with feature selection via mutual information (MI). 5-fold stratified cross-validation; RF compared against SVM, KNN, Gaussian Naive Bayes.	Plasma / serum–derived exosomes: 205 cancer and 51 control samples from 5 independent studies (Hoshino 2020, Vykoukal 2017, Li 2021, Lin 2022, Hallal 2020). Cancers: breast, colorectal, glioblastoma, lung carcinoma, liver, neuroblastoma, pancreatic.	From 46 proteins common across the 5 plasma/serum datasets, top features were ranked by MI. Best performance at 18-protein panel. RF with these 18 proteins (5-fold CV): AUROC = 0.96, accuracy = 0.92, precision = 0.94, recall = 0.96. On an independent test set, RF achieved AUROC = 0.99, accuracy = 0.95, precision = 0.96, recall = 0.98, with only 1 misclassified sample and 51/51 cancer samples correctly classified. RF clearly outperformed SVM, KNN, and Gaussian NB.	46 overlapping proteins across 5 plasma/serum exosome studies; MI-based feature selection identified 18-protein cancer exosome signature. Top-ranked feature: APOC1, decreased in cancer vs. controls and previously reported downregulated in multiple cancers. Other features include proteins with known cancer links (e.g., ITIH3, etc., discussed in the paper).	[202]
Classification of 5 common cancer types (breast, colorectal, glioma, lung, pancreatic) using plasma/serum exosomes	Random Forest classifier with MI-based feature selection. 5-fold stratified CV; independent test set (40% of samples) to reduce overfitting.	Plasma / serum exosomes from patients with: breast cancer, colorectal cancer, glioma, lung cancer, pancreatic cancer (subset of the plasma/serum cohort).	46 proteins shared across plasma/serum datasets were ranked by MI for multiclass classification. RF performance vs. number of features showed optimum at 5 proteins. With these 5 proteins: 5-fold CV on 60% training set to accuracy = 0.99; independent test on remaining 40% to accuracy = 0.94. Protein abundances showed cancer-type–specific patterns (e.g., HRG enriched in colorectal cancer exosomes, aligning with prior evidence of HRG promoting CRC migration).	5-protein panel (names listed in Fig. 5 and associated data files) selected from the 46 overlapping plasma/serum exosome proteins via MI + RF AUROC criterion. These proteins collectively encode type-specific exosome signatures for the five cancers.	[202]
Pan-cancer detection from urine-derived exosomes (urologic + non-urologic cancers)	Random Forest classifier with MI-based feature selection; AUROC used as main metric; 5-fold stratified CV + independent test.	Urine-derived exosomes: 261 cancer and 124 control samples from 4 studies (Dhondt 2020, Zhang 2018, Øverbye 2015, Suh 2022). Cancer types included bladder, prostate, renal, lung, cervical, colorectal, esophageal, gastric, etc.	From 229 proteins shared across the 4 urinary datasets, MI ranking + RF performance vs. feature count gave optimum at 17 proteins. With this 17-protein panel, RF (5-fold CV): AUROC = 0.96, accuracy = 0.90, precision = 0.92, recall = 0.93. On independent test set: AUROC = 0.91, accuracy = 0.82, precision = 0.83, recall = 0.92. These results demonstrate that urinary exosome proteomics can detect both urologic and non-urologic cancers with high diagnostic performance.	229 overlapping urinary exosome proteins; MI-selected 17-protein urinary cancer exosome signature (names in Fig. 6 and source data). Most of these proteins show strong differential abundance between cancer and control in heatmaps, suggesting pathophysiological relevance for tumor-associated changes in the urogenital axis and beyond.	[202]
Single-vesicle multi-miRNA profiling for cancer diagnosis and cancer-type classification using EV miRNA signatures	Convolutional neural network (CNN)–based deep learning model on single-EV TIRF images; softmax output, cross-entropy loss, ReLU + dropout, early stopping; compared against Bayes, Decision Tree, KNN, and SVM (all ML baselines < 50% accuracy)	EVs from 5 cancer cell lines (A549 lung, HeLa cervical, HepG2 liver, LoVo colon, MCF-7 breast) and normal plasma; clinical plasma EVs from 20 cancer patients (5 lung, 5 breast, 5 colon, 5 cervical) and 5 healthy controls; triple-positive EV subpopulation defined by miR-21/miR-122/miR-375	Triple-positive EVs (miR-21/122/375) are the most informative subpopulation; CNN on triple-positive EV images achieves > 95% classification accuracy for single EVs from 6 sources (precision 0.96 ± 0.03, recall 0.95 ± 0.04, F1 0.96 ± 0.03, ACCavg 0.95 ± 0.04); EV-level cancer vs. normal discrimination accuracy ≈ 85%; in a 25-subject clinical cohort, per-patient cancer diagnosis and 4-cancer-type classification reached 100% overall accuracy, clearly outperforming classical ML models	Total internal reflection fluorescence (TIRF) microscopy with nanoflare DNA probes + catalytic hairpin assembly (CHA) amplification for in situ detection of miR-21, miR-122, miR-375 in single EVs; GA-functionalized coverslips for unbiased EV capture; RGB TIRF images cropped to 20 × 20-pixel single-EV patches; ~10,000 single-EV images used for DL (train/val/test = 6:2:2); t-SNE for feature visualization	[203]
EV protein–based liquid biopsy for metastatic breast cancer (MBC): diagnosis (MBC vs. NMBC vs. healthy), monitoring treatment response, and predicting progression-free survival	Linear Discriminant Analysis (LDA) to build multivariate EV “signatures”: EVDX (diagnostic), EVM (treatment response, using ΔIntensity), EVP (prognosis); multivariate linear regression (EVTS) to approximate tumor burden; logistic regression compared but similar or slightly inferior to LDA	Plasma-derived EVs from breast cancer patients and healthy donors: 36 MBC, 21 non-metastatic BC (NMBC), 66 healthy donors for diagnosis; 78 MBC samples (training/validation) + 35 prospective samples for response monitoring; 59 MBC patients (baseline EVs) + 16 prospective MBC patients for PFS prediction	EVDX (weighted sum of 8 EV proteins: CA 15 − 3, CA 125, CEA, HER2, EGFR, PSMA, EpCAM, VEGF) achieved AUPRC 0.9912 (BC vs. HD) and 0.9433 (MBC vs. NMBC), with 91.1% overall accuracy across MBC/NMBC/HD; EVTS (multivariate regression of 8 markers) correlated strongly with tumor size (R² ≈ 0.80 in MBC, ≈ 0.99 in NMBC); EVM (LDA on ΔIntensity of 8 markers) classified PD vs. PR/SD with AUC ≈ 0.94 and accuracy 88.9% (training), AUC ≈ 0.91 and accuracy 87.9% (validation), and 85.2% accuracy in a prospective cohort; EVM tracked longitudinal treatment response better than plasma CA 15 − 3 (~ 88.6% concordance vs. 62–80% for CA 15 − 3); EVP (baseline LDA signature) predicted PFS (median 475 vs. 254 days, HR ~ 4.1 univariate and ~ 6.4 multivariate), outperforming CA 15 − 3; EV PSMA alone was also a significant independent prognostic marker	Thermophoretic aptasensor (TAS) with Cy5-labeled aptamers targeting 8 EV surface proteins, using size-dependent thermophoretic enrichment (> 10³-fold) of EVs (~ 10¹⁰–10¹¹ mL⁻¹) from 1 µL plasma; ultrasensitive EV detection with LoD 3.8 × 10⁷ mL⁻¹ (> 10²-fold more sensitive than ELISA); nanoparticle tracking analysis (NTA) and TEM for EV characterization; standard clinical imaging (CT/MRI) and CA 15 − 3 used as comparators	[204]
Noninvasive monitoring of cell cycle stages and mitotic subphases, and probing molecular mechanisms via exosomal vibrational fingerprints	Dimensionality reduction and classification with Linear Discriminant Analysis (LDA); supervised classification with Support Vector Machine (SVM) trained on full SERS spectra (compared against KNN and XGBoost, which performed worse); PCA and t-SNE explored but LDA gave best separation	Exosomes (30–150 nm) secreted from synchronized HeLa cells arrested at G0/G1, S, G2/M phases (serum starvation, double-thymidine block, nocodazole) and at mitotic subphases (prometaphase, metaphase, anaphase/telophase using nocodazole, MG-132, blebbistatin); comparative exosomes from normal cervical epithelial H8 cells vs. HeLa at metaphase; exosome concentration and size confirmed by NTA and TEM	Label-free exosomal SERS spectra plus LDA cleanly separated exosomes from three cell cycle stages with 90% confidence ellipses; SVM model trained on exosomal SERS achieved ~ 80.5% mean accuracy on held-out test data (≈ 95% for G0/G1, 81.8% for S, 64.7% for G2/M; ROC AUCs 0.98, 0.87, 0.84 respectively); when applied to normally cultured HeLa cells and longitudinal samples (24 h vs. 65 h), SVM correctly inferred dominant cell-cycle stage with accuracies up to 95.5% (G0/G1-rich) and ~ 80–90% for dynamic time-point analysis (average ~ 85%); SERS-based mechanistic analysis showed loss of 503 cm⁻¹ disulfide band (–S–S–) across mitosis, increased phenylalanine bands (998, 1592to1598 cm⁻¹) and validated rise in Phe content by HPLC, and subtle shifts in tryptophan band (1359 cm⁻¹) consistent with microtubule/protein conformational changes; comparison of HeLa vs. H8 exosomal spectra at metaphase revealed higher sulfur-containing amino acid and Phe signatures in HeLa, coexistence of α-helix and β-sheet in HeLa vs. predominantly α-helix in H8 (amide III bands 1235/1284 cm⁻¹), suggesting altered protein folding/structure in cancer cells	Label-free surface-enhanced Raman spectroscopy (SERS) of exosomes on a dense monolayer Ag nanoparticles nanomembrane (AgNPs-NM) prepared by surface-tension–gradient (Marangoni) self-assembly, providing uniform high-density electromagnetic “hot spots”; confocal Raman microscopy at 532 nm (0.24 mW, 5 s), with preprocessing via Savitzky–Golay filtering, airPLS background removal, and Min–Max normalization; supporting characterization by TEM, NTA, flow cytometry, confocal fluorescence imaging, FDTD field simulations, and HPLC amino acid quantification	[205]
EV-mediated metastasis & microenvironment priming in colorectal cancer (CRC); quantitative tracking of CRC-EV trafficking in colon and lung	Dual U-Net–based deep learning segmentation and tracking pipeline for fluorescence microscopy time-lapse images. Triple-prediction strategy (nuclei, EVs, merged channel) with fusion postprocessing; distance-transform regression branch + categorical branch with Dice loss and smooth L1 loss; data augmentation (flip, contrast, scaling, blur/noise) to robustly segment cells, nuclei, and EVs, then compute EV count per cell and EV area coverage over time.	EVs isolated by differential ultracentrifugation from human colorectal cancer cell line Colo-320DM (CRC-EVs), characterized as exosome-like vesicles (~ 100–140 nm, negative ζ-potential, ALIX/HSP90α/ACTB+, GM130–, CD63+/CD81+). Tested on 2D cultures of parental CRC cells (Colo-320DM), healthy colon fibroblasts (CCD-18Co), lung fibroblasts (MRC-5), lung epithelial cells (A549), plus 3D bioprinted colon tubes (CCD-18Co ± Colo-320DM) and 3D lung ALI model (PCL-Gel scaffold with MRC-5 + A549).	CRC-EVs are non-cytotoxic at 10–50 µg/mL up to 72 h and are internalized by nearly 100% of cells across conditions; however, uptake is strongly cell-type–specific. • Flow cytometry + DL tracking show preferential EV uptake by nonmalignant fibroblasts (MRC-5 > A549 > CCD-18Co ≫ Colo-320DM), indicating strong tropism toward stromal and healthy cells rather than parental tumor cells. • Deep learning–derived metrics (EV count per cell, EV area %) recapitulate flow cytometry trends, validating the model as a quantitative EV-tracking tool. • In 3D colon constructs, CRC-EVs preferentially accumulate in fibroblast-rich outer regions and minimally in tumor cell–rich lumen, suggesting fibroblast conditioning of the colon microenvironment. • In 3D lung ALI models, CRC-EVs penetrate the tissue from the “vascular” side and are taken up by both A549 epithelium and MRC-5 fibroblasts, supporting a role in lung premetastatic niche formation and metastatic tropism. • Overall, the study positions CRC-EVs as active mediators of stromal reprogramming and metastasis, and the DL pipeline as a generalizable tool for high-content EV trafficking analysis.	Time-lapse spinning-disk fluorescence microscopy (2D and 3D; multichannel imaging of nuclei, membranes, EVs). Deep CNN (Dual U-Net) segmentation with custom loss and fusion postprocessing; EV tracking and per-cell quantification. • Standard EV characterization: NTA, DLS, ζ-potential, Bradford protein assay, TEM, Western blot (EV and non-EV markers), bead-based flow cytometry for CD63/CD81. • 3D bioprinting of colon-like tubes (gelatin–alginate bioink + CCD-18Co) and electrospun PCL–gelatin lung scaffolds under air–liquid interface culture.	[206]
Single-EV morphological profiling and architectural heterogeneity mapping	Custom deep neural network–based segmentation pipeline (Cellpose 2.0–derived “EVpose” model; expert-in-the-loop transfer learning; U-Net–style architecture with residual blocks; automated feature extraction of eccentricity, equivalent diameter, major/minor axes)	EVs from multiple origins: tumorigenic cell lines (e.g., Panc1, T3M4), non-tumorigenic/immortalized cell lines (HPNE, HEK293T), and human serum; EVs isolated by differential ultracentrifugation, size-exclusion chromatography, and OptiPrep density gradient	Cryo-TEM + EVpose segmentation of 7,576 individual EVs revealed robust architectural heterogeneity across sources and isolation methods, with three dominant classes (single spherical, tubular/rod-like, double/multilamellar vesicles). Average eccentricity 0.5366 ± 0.2 and equivalent diameter 132.43 ± 67 nm indicate that native EVs are predominantly non-spherical. Morphological diversity (shape/size) is intrinsic, conserved in biofluids, and largely independent of isolation approach or − 80 °C storage. Automated pipeline achieved ~ 95% segmentation accuracy with only 32 training images and processed 669 cryo-TEM micrographs (~ 7,500 + EVs) in ~ 40 min, enabling scalable quantitative single-EV morphology analysis.	High-resolution cryogenic transmission electron microscopy (cryo-TEM) of vitrified EVs; automated segmentation and morphometric analysis using custom Cellpose-based neural network (EVpose); extraction of per-EV structural metrics (eccentricity, diameter, major/minor axes); comparison across isolation methods (SEC, dUC, DG) and fresh vs. freeze–thaw conditions	[207]

AI: artificial intelligence, ACCavg: average accuracy, APTES: (3-aminopropyl)triethoxysilane, AUC: area under the curve, AUROC: area under the receiver operating characteristic curve, AUPRC: area under the precision–recall curve, AuNP: gold nanoparticle, BC: breast cancer, BCC: benign colorectal disease (BCD) – benign colorectal disease, BCLC: Barcelona Clinic Liver Cancer (staging system), BP neural network: backpropagation neural network, CAP1: adenylyl cyclase-associated protein 1, CEA: carcinoembryonic antigen, CE: classification error, CHA: catalytic hairpin assembly, CLTC: clathrin heavy chain, CNN: convolutional neural network, CRC: colorectal cancer, cryo-TEM: cryogenic transmission electron microscopy, CT: computed tomography, Cy5: cyanine-5 fluorophore, DFT: discrete Fourier transform, DG: density gradient, DIA-MS: data-independent acquisition mass spectrometry, DIA-NN: data-independent acquisition–neural network, dUC: differential ultracentrifugation, DL: deep learning, DLS: dynamic light scattering, DSPE: 1,2-distearoyl-sn-glycero-3-phosphoethanolamine, EGFR: epidermal growth factor receptor, ELISA: enzyme-linked immunosorbent assay, EpCAM: epithelial cell adhesion molecule, EV: extracellular vesicle, EVs: extracellular vesicles, EVDX: EV-based diagnostic signature, EVM: EV-based monitoring signature, EVP: EV-based prognostic signature, EVTS: EV-derived tumor score (EV tumor burden score), FFT: Feature Fusion Transformer, FDTD: finite-difference time-domain, GA: glutaraldehyde, G0/G1, S, G2/M: cell cycle phases (gap 0/1, synthesis, gap 2/mitosis), GSEA: gene set enrichment analysis, HCC: hepatocellular carcinoma, HD: healthy donor, HEK: human embryonic kidney (cell line), HEK293T: human embryonic kidney 293T cell line, HPNE: human pancreatic nestin-expressing (cell line), HR: hazard ratio, HRG: histidine-rich glycoprotein, KNN: k-nearest neighbors, Lasso: least absolute shrinkage and selection operator, LDA: linear discriminant analysis, LLM: large language model, LoD: limit of detection, MBC: metastatic breast cancer, MIL: multiple instance learning, MI: mutual information, MPLS: multiplicative polynomial least squares (baseline correction), MRI: magnetic resonance imaging, MSN: moesin, NB: Naive Bayes, NTA: nanoparticle tracking analysis, NSCLC: non-small-cell lung cancer, OPLS-DA: orthogonal partial least squares discriminant analysis, PCA: principal component analysis, PCL: poly(ε-caprolactone), PF4: platelet factor 4, PFS: progression-free survival, PRC/PR curve (in AUPRC/PRAUC): precision–recall curve, PRAUC: area under the precision–recall curve, PSMA: prostate-specific membrane antigen, RAG: retrieval-augmented generation, RF: Random Forest, ReLU: rectified linear unit, RNA-seq: RNA sequencing, ROC: receiver operating characteristic, S: synthesis phase (cell cycle), scRNA-seq: single-cell RNA sequencing, SEC: size-exclusion chromatography, SERS: surface-enhanced Raman spectroscopy, SHAP: Shapley additive explanations, SP3: single-pot, solid-phase–enhanced sample preparation, SVM: support vector machine, TAS: thermophoretic aptasensor, TCGA: The Cancer Genome Atlas, TEM: transmission electron microscopy, TIRF: total internal reflection fluorescence, TNM: tumor–node–metastasis (staging system), TOO: tissue of origin, TSG101: tumor susceptibility gene 101, U-Net: U-shaped convolutional neural network, VEGF: vascular endothelial growth factor, WB: Western blot

## Applications of ML in EV research

### Disease diagnosis and prognosis

The use of EVs as non-invasive biomarkers for prognosticating and diagnosing diseases is advancing rapidly, owing to their stability in biofluids, molecular specificity, and capacity to transfer real time physiological information [208]. Coupled with ML, the examination of EVs is currently generating diagnostic platforms with high accuracy, scalability, and low invasiveness for diverse pathological states [209], including oncology, NGDs [210], and inflammatory diseases [211, 212].

**Fig. 2:**
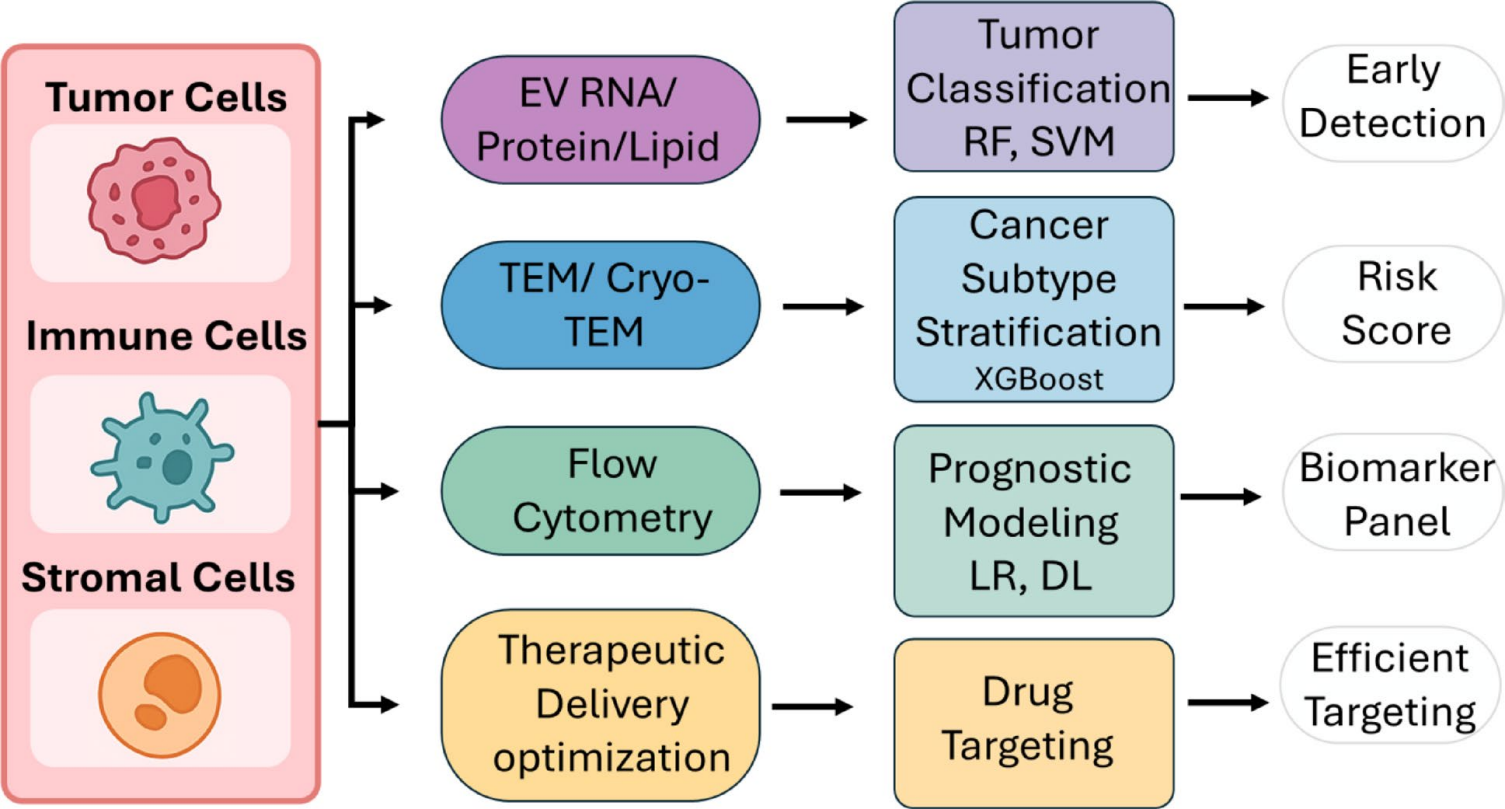
AI assisted with the fusion of extracellular vesicle data modalities in cancer research. This figure shows the convergence of biological sources, EV analytical modalities, ML approaches, and translational outputs in the realm of cancer. EVs secreted by tumor cells, immune cells, and stromal cells have diverse molecular cargo

#### EVs as liquid biopsy tools

EVs are secreted into almost all bodily fluids and carry encapsulated materials that mirror the properties of cells that produced them, including proteins, RNAs (particularly miRNAs), lipids, and DNA pieces [213]. These properties make EVs particularly attractive for “liquid biopsy” applications that enable real time molecular diagnostics (Fig. 2) free from the need for invasive tissue biopsies [214]. However, the heterogeneity and complexity of EV cargos require advanced computational modeling, an area where ML has proven to be adept in feature selection, pattern detection, and predictive modeling [215].

#### ML driven cancer diagnostics: from blood to stool derived EVs

Colorectal cancer (CRC), identified as one of the leading causes of cancer deaths worldwide, shows significantly enhanced survival when detected early [216]. Current diagnostic modalities, including colonoscopy, carcino embryonic antigen (CEA), and carbohydrate antigen 19 9 (CA19 9), show low sensitivity, especially in early stages of the disease [217, 218]. A recent study by Zhang et al. presented an important, groundbreaking ML assisted platform called Fecal Extracellular Vesicle microRNA Signatures (FEVOR) for intrusive CRC detection. The experiment procedure begins with an miRNA assessment of EVs separated from fecal specimens, the creation of a Clustered Regularly Interspaced Short Palindromic Repeat (CRISPR)/Cas13a based diagnosis system, and its pairing with supervised ML categorizers for diagnostic modeling. For a group of 38 CRC patients, the ML model identified 37 cases with full precision accuracy of 97.36%, thereby outdoing the CEA (26.3%) and CA19 9 (7.9%) conventional biomarkers [219].

**Fig. 3:**
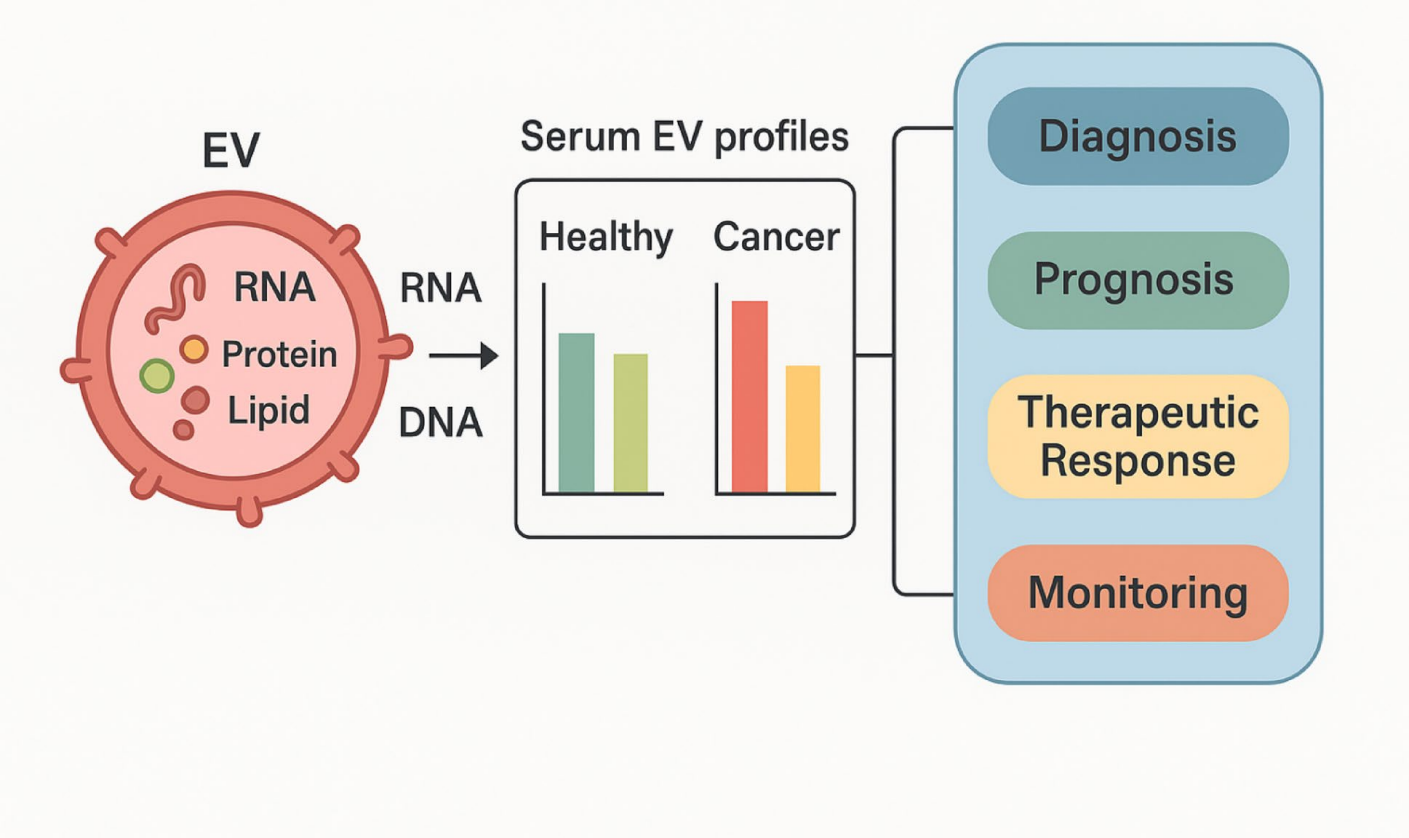
EVs as cancer biomarkers, EV-derived lipid, protein, and RNA profiles are unique in healthy versus cancer conditions and enable applications in disease monitoring, prediction of response to therapy, prognosis, and diagnosis

In addition, a longitudinal study of pre and postoperative patients with CRC and colorectal adenomas (CAs) emphasized the potential of tumor markers for surveillance and early disease detection [220]. This indicates that panels of EV-derived miRNAs can be useful for diagnosis and also for risk assessments and prognostic studies (Fig. 3). A recent study showed the convergence of molecular developments, CRISPR biosensing technology, and ML integration in EV diagnostics and is set to drive translational progress in stool based cancer screening methods [221].

#### Serum EVs in pancreatic and prostate cancers

In pancreatic cancer, ML models trained on serum EV miRNAs (e.g., miR 21 and miR 1246) outperformed CA19 9 by achieving AUCs of > 0.93, showing promise for asymptomatic early stage detection [222]. Similarly, in prostate cancer, the EVMAP platform applied XGBoost models to multivariate flow cytometric EV data, surpassing PSA screening by accurately detecting high grade tumors (Gleason score ≥ 3) and integrating clinical metadata for personalized risk estimations [114].

#### Applications in neurodegeneration and sepsis

In NGDs such as amyotrophic lateral sclerosis (ALS), ML analysis of individual genes (e.g., *ANXA5* and *GPM6A*) enabled the differentiation of early and late stage patients from NGDs [223]. Similarly, in sepsis, a support vector machine (SVM) trained on Raman signals from plasma derived EVs achieved 97.5% sensitivity and 90.0% specificity, demonstrating the potential of EV signal identification for diagnosis and etiological classification [224].

#### Translational medicine and validation of cohorts

The relevance of EV-based ML models in clinical applications depends on their validation in multicenter trials. With strict evaluations, the functionality of a model can be guaranteed across different settings and populations. On one hand, Kellner et al. [225] employed an advanced biosensor CRISPR based biosensing platform with exceptional sensitivity and specificity for detecting nucleic acids [226]. This showed how biosensors can be combined with CRISPR technology for point of care testing. On the other hand, Bhaiyya et al. [227] recommended the use of ML enhanced biosensors to increase the accuracy and frequency of point of care tests. According to them, ML can help interpret complex data and make clinical decisions. In general, those authors thought it possible to develop a real time robust diagnostic device that is appropriate for various settings by combining EV analysis, ML, and advanced biosensors.

### Understanding EV biology and function

**Fig. 4:**
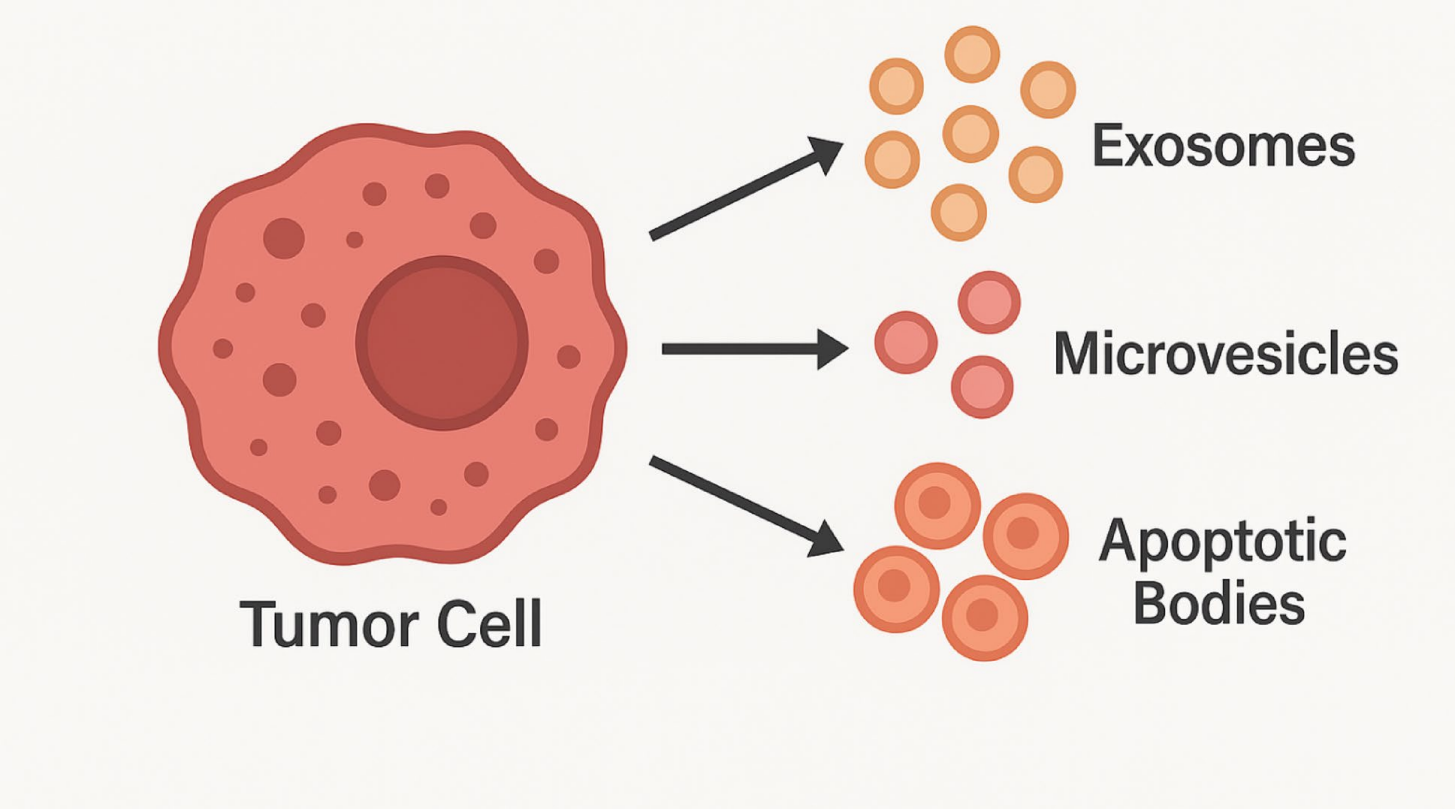
Simplified biogenesis of tumor derived EVs, Tumor cells release distinct EV subtypes including exosomes, microvesicles, and apoptotic bodies each with unique biogenetic pathways and functional roles in cancer progression

EVs are now identified as having high promise in diagnostic [85] and therapeutic modalities; yet, understanding their biological origins (Fig. 4), molecular mechanisms, and physiological functions remains an uphill battle. This is mainly due to the heterogeneity of EV biogenesis pathways, specificity of cargo loading into EVs [5], and the complexity of intercellular communication mediated by vesicles. Advances in multi omics profiling, coupled with ML approaches, are now augmenting our ability to delineate mechanistic aspects of EV biology with incredible accuracy [228].

#### Deciphering EV biogenesis pathways via ML

Building on the conventional division of EVs into exosomes, microvesicles, and apoptotic bodies described in Sect. 1.1, here we focus on how ML-driven analyses can refine and even challenge these categories. By integrating high-dimensional information on EV size, surface markers, cargo profiles, and biophysical properties, ML models can uncover latent EV subpopulations and biogenetic trajectories that are not captured by classical morphology- or size-based classification schemes [115, 229].

To meet this challenge, ML approaches leveraging high dimensional proteomic and lipidomic datasets have been used to classify EV subtypes based on markers linked to biogenesis [116]. Unsupervised clustering methods, such as k means and hierarchical clustering, applied to EV protein signatures have identified concealed subpopulations of vesicles enriched in ESCRT dependent proteins like tumor susceptibility gene 101 (TSG101) and Alix or the CD9 and CD81 tetraspanins. This strategy allows for EV classification based on data driven information beyond conventional morphological criteria [230].

In addition, supervised learning based approaches, particularly using RFs and XGBoost, have been used to determine EV origins (e.g., discriminating between endosomal versus plasma membrane derived origins) by identifying distinctive molecular signatures in different vesicle populations [231]. Such predictive models are informative and serve as hypothesis generating tools that can guide experimental verification of proposed biogenetic pathways [44, 232].

#### Uncovering cargo sorting mechanisms

As summarized in Sect. 1.1, EV cargos comprise diverse biomolecules, including miRNAs, mRNAs, DNA fragments, lipids, and proteins that mirror the physiological or pathological state of the cell of origin [233]. Rather than reiterating these basic cargo classes, in this subsection we emphasize how ML methods leverage this rich cargo space to infer selective packaging rules, disease-associated signatures, and functional EV phenotypes.

ML based feature selection and classification methods can be used to identify sequence motifs and secondary structures of miRNAs that are responsible for their selective packaging into EVs. For instance, studies using SVMs and least absolute shrinkage and selection operator (LASSO) regression approaches can identify high occurrence of GGAG and GCAC motifs in EV associated miRNAs, implicating the RNA binding proteins hnRNPA2B1 [234] and YBX1 [235] in the sorting process.

In addition, DL models, such as autoencoders (AEs) and variational AEs (VAEs), can be used to reduce the dimensionality of multi-omics data to allow the discovery of latent variables correlated with cargo loading efficiency and vesicle classes. These models can be used to investigate nonlinear relationships among proteins, RNAs, and lipids, which can reveal co packaging events or functional relationships of EVs [236, 237].

#### Multi omics integration for functional profiling

To grasp the full picture of EV function, a mix of omics fields is required, combining EV transcriptomics, proteomics, and lipidomics to determine cross modality correlations, for instance, linking miRNAs within EVs to their target proteins or linking lipid species with their transport and uptake abilities [238, 239].

Extensive research employed hypoxia exposed tumor cells versus EVs of hypoxia exposed tumor cells. Such a structured model revealed miR 210, along with ceramide lipids, to be co enriched in hypoxic EVs, providing additional evidence for the assertion that co regulation of EV cargos can help tumors adapt to hypoxia [240]. Follow up work can utilize graph structured ML methods, such as network propagation and graph neural networks (GNNs), to model molecular interactions that exist in EV cargos and define the regulatory modules associated with vesicle function, e.g., angiogenesis and immune modulation^[239]^. These approaches can provide an integrated view of how EVs modulate signaling pathways in target cells.

### QC in EV isolation and characterization

The potential of EVs for therapeutic and diagnostic uses [183] depends not only on their molecular composition, but also on the reproducibility, reliability, and purity of methods of their extraction and characterization. Given EV heterogeneity in terms of size, biogenesis, and cargo, it is necessary to come up with protocols for their extraction that will help standardize the use of vesicles in further studies [113]. Parameters of techniques used for EV isolation, such as ultracentrifugation, size exclusion chromatography (SEC), immunoaffinity capture, and microfluidic methods, can greatly vary and, in turn, affect the composition, concentration, or functional activity of vesicles [241]. This may become a major problem in reproducing previous study results and moving them into clinical practice [69].

In the recent past, ML methods were incorporated into workflows for EV QC, and they showed promise in terms of optimizing isolation protocols, classifying EV subtypes, and detecting inconsistencies in production batches of clinical grade EVs [242].

#### ML assisted assessment of isolation techniques

Differential ultracentrifugation is one of the most widely used methods for isolating EVs, with the advantage of scalability; however, this approach is also simultaneously defined by the co isolation of contaminants such as protein aggregates and lipoproteins [243]. In contrast, SEC and immunoaffinity based techniques yield higher purities at the expense of scalability and yield. Separating EV enriched fractions from contaminating fractions often requires time consuming biochemical assays [244].

ML algorithms, particularly unsupervised clustering and anomaly detection models, have been applied to multi parametric datasets generated during isolation, including NTAs, protein concentrations, zeta potentials, and flow cytometric profiles. For instance, k means clustering and hierarchical algorithms have been used to group EV preparations by isolation method, revealing distinct compositional fingerprints associated with each technique [245].

In recent developments, supervised ML algorithms were designed to predict the best isolation strategies specific to certain downstream applications, e.g., proteomics and therapeutic interventions, based on the sample type and origin [246]. A research study by Waury et al. using RF classifiers integrated biochemical features from EV samples to suggest EV associated proteins, thus improving the yield purity ratio [247].

#### Ensuring consistency of clinical grade EVs

As EVs move into clinical trials for uses such as diagnostics, drug delivery systems [248], or regenerative therapies, consistency from batch to batch, sterility maintenance, and identity verification are essential. Regulatory agencies are beginning to develop release standards for EV-derived products that include measurements of the particle size distribution, concentration, purity, and surface marker expressions [186].

ML tools are becoming well known as valuable real time monitoring tools in biomanufacturing processes. As an example, combining flow cytometry based characterization of EVs with XGBoost classifiers enabled the automated detection of abnormalities in marker expression profiles that indicate suboptimal culture conditions or problems with isolation [249]. In a similar fashion, the use of time series anomaly detection with recurrent neural networks (RNNs) has potential to identify changes in EV yields or quality during bioreactor operations [250]. The International Society for Extracellular Vesicles (ISEV) promotes careful documentation of variables linked with the preparation of EVs through the efforts of publications like the MISEV2018 [251] and the most recent MISEV2023 guidelines [252]. Combining ML algorithms with these developed standards can improve the automation of compliance checks, enhance data consistency, and facilitate predictive modeling in the context of the effectiveness or functionality of EVs [253].

Nonetheless, ML enabled QC systems promise to bridge the gap between academic EV research and good manufacturing practice (GMP) compliant manufacturing, enabling the development of reproducible, scalable, and clinically robust EV-based products [254].

### Drug delivery and therapeutic applications

EVs are increasingly recognized as natural nanocarriers for drug delivery, due to their biocompatibility, low immunogenic reaction, stability in the bloodstream, and intrinsic ability to cross biological barriers, such as the blood brain barrier (BBB). The endogenous nature and intrinsic targeting capability of EVs offer a huge advantage over artificial NPs, making EVs flexible carriers for targeted drug delivery, gene therapy, and immunomodulation [255–257].

Despite these advantages, challenges persist that prevent the realization of reproducible loading efficiencies, accurate tissue targeting, scalable manufacturing processes, and customized delivery approaches. Recent advances in ML have accelerated efforts to surmount these challenges by facilitating the systematic design and optimization of therapeutics derived from EVs, thereby enabling the development of targeted, cell type specific, and functionally tailored EVs [258, 259].

#### ML for optimization of tissue targeting

EV tropism, their natural tendency to accumulate in specific tissues, is influenced by surface proteins, lipids, and size. However, native EVs often lack the targeting precision needed for therapeutic delivery, especially in complex diseases such as cancer or neurodegeneration. ML approaches, particularly supervised classification models and predictive regression algorithms, are now being employed to map and optimize EV tissue interactions [260]. Similarly, reinforcement learning based frameworks have been established to incrementally optimize the process of picking targeting ligands (e.g., peptides and aptamers) that can be genetically or chemically altered on the surface of EVs to enhance their binding to tumor microenvironments (TMEs) or inflamed tissues [261].

#### ML assisted design of engineered EVs for precision medicine

**Fig. 5:**
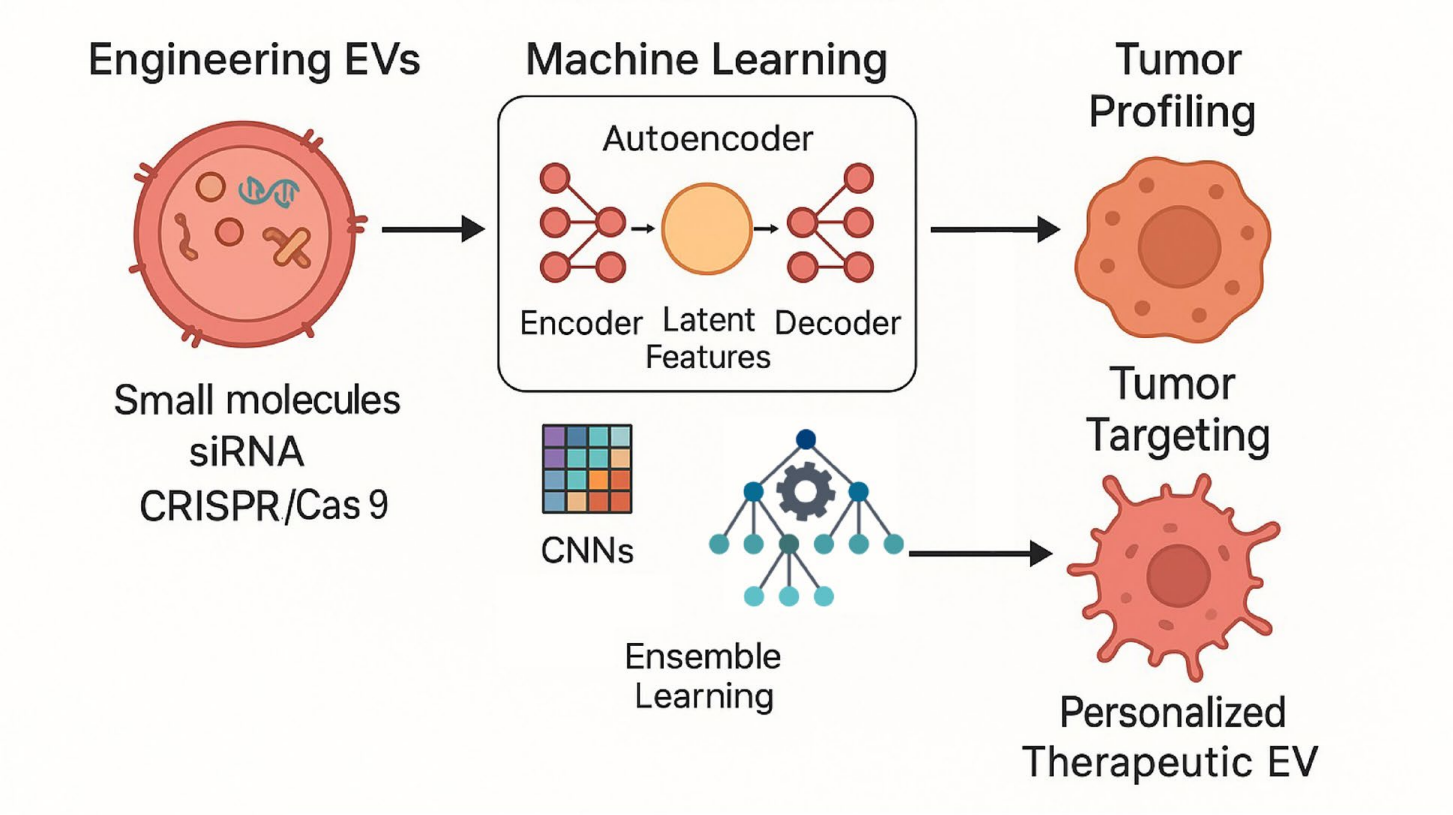
ML designed engineered EVs for precision medicine, Machine learning techniques, i.e., autoencoders, CNNs, and ensemble learning, guide the optimization of therapeutic EVs enriching cargo loading, targeting, and personalization by tumor profiling for targeted cancer therapy

Engineering EVs for therapeutic payload delivery ranging from small molecules and small interfering (si)RNAs to CRISPR/Cas systems requires optimization of cargo loading, release kinetics, stability, and functional targeting [262, 263]. ML models can inform each of these stages by integrating high dimensional experimental data (Fig. 5) (e.g., NP tracking, zeta potential, RNA Seq, and lipidomics) [264].

In future research, DL frameworks based on AEs can be utilized to detect latent features that predict EV loading capacities and release dynamics for different cell types, as well as loading strategies (e.g., electroporation, sonication, and lipid fusion). These features are important when choosing appropriate donor cells or for modifying biogenesis pathways to improve the retention of therapeutic payloads.

Also, an AI guided EV design platform can be developed by combining CNNs and ensemble learning to engineer exosome mimetic NPs that encapsulate drugs and are decorated with targeting peptides for cancer therapy. The system may exhibit enhanced tumor accumulation and reduced off target toxicity in animal models, compared to traditional liposomal delivery.

In addition, the use of ML platforms is increasingly common in the tailoring of EV-based therapies by linking personalized molecular profiles (e.g., tumor transcriptomes and immune signatures) with ideal EV cargo compositions and targeting strategies. These strategies are enabling the development of digital twins for precision nanomedicine, where computational modeling guides the design of in vivo therapeutic strategies.

#### AI in clinical translation and drug repurposing via EVs

ML frameworks have also been leveraged for drug repurposing [265], using EVs as carriers. For instance, deep neural networks (DNNs) can be trained on transcriptomic responses to EV delivered agents to identify synergistic drug combinations in chemoresistant cancers, guiding rational combinatorial therapy design [266].

The integration of ML into manufacturing systems is improving engineered vaccine manufacturing processes to ensure batch to batch consistency, forecast efficacy, and create flexible process control that meets GMP requirements, thus enabling the regulatory approval processes for engineered vaccine therapies.

## Challenges in ML and EV research

### Standardization of EV isolation and characterization

Despite quick progress, using ML in EV studies has some recurring issues in experimental design, data quality, model making, and putting findings into practice. These issues can be grouped into four areas: (i) making EV isolation and characterization uniform, (ii) dealing with EV variety, (iii) understanding ML models, and (iv) combining different types of data [267, 268].

#### Variability in isolation techniques

Commonly used techniques for EV isolation include differential ultracentrifugation (dUC), a commonly used method that involves multiple steps of centrifugation. Although the method can be scaled, it often results in the co purification of non exosomal contaminants, including protein aggregates and lipoproteins, as well as batch to batch variation [269]. Size exclusion chromatography (SEC) offers superior EV purity and is gentle on vesicles, but typically has lower yields and variable recovery rates depending on the column type and sample volume [270]. Density gradient centrifugation, ultrafiltration, and precipitation using polymers all have distinct advantages and limitations with regards to purity, recovery efficiency, and scalability. Immunoaffinity capture enables specific enrichment of EV subpopulations via antibodies (e.g., anti CD63), but often excludes biologically relevant, marker negative vesicles and is cost prohibitive at scale [271].

Differences in buffer composition, type of rotor, spin duration, temperature, and filtration conditions add to the complexity, leading to non-standardized populations of EVs, even when the same isolation method is used [272].

#### Impacts on data reproducibility and consistency

The absence of consensus protocols means that there is significant between laboratory variability, which hampers the reproducibility of EV-based biomarkers, functional assays, and therapeutic efficacy studies. Additionally, meta analyses of EV omics datasets often reveal clustering based on methodological differences instead of biologically relevant classes, which suggests that technical artifacts prevent precise biological interpretation [273].

These inconsistencies in approaches are a major challenge for:

- biomarker discovery, where small differences in isolation can skew concentration and cargo composition [274];
- clinical trials where batch variability affects efficacy, safety, and regulatory approval [275]; and.
- applications of ML, in which non standardized training data can produce noise and lead to model overfitting or poor generalization over different datasets [276].

#### Toward standardization: minimal information for studies of extracellular vesicles (MISEV) guidelines and ML integration

To address these challenges, the MISEV2023 guidelines set by the International Society for Extracellular Vesicles (ISEV) provide critical guidelines on the reporting of isolation methods, QC, and experimental reproducibility. The latest version, MISEV2023, highlighted improved standards for data annotation, the application of orthogonal quantification methods, and the development of validation metrics [23].

ML can play a critical role in enabling standardization, by:

- quantifying inter-method variability using unsupervised clustering of EV characterization data (e.g., size distribution, surface markers, and cargo contents);
- classifying provenances of isolation methods according to multi parametric EV profiles, thus controlling method specific biases in downstream analyses.
- detecting batch effects and standardizing data across platforms using domain adaptation or transfer learning methods; and.
- improving protocol design by modeling the isolation output quality (e.g., purity and functional capacity) as a function of input conditions and experimental parameters.

In a very recent study, ML models trained with flow cytometric data and a unique set of biomarkers (CD45, CD63 and EphA2) effectively predicted the presence of pancreatic tumors of different natures (exocrine/endocrine) in patients’ plasma with greater than 90% accuracy. This discovery revealed subtle, yet consistently discriminatory signatures of each method in EV profiles [277].

In particular, these ML strategies are well suited to harmonizing data from ultracentrifugation-, SEC-, and microfluidics-based preparations by modeling and correcting isolation-induced shifts in EV size distributions, surface-marker patterns, and cargo profiles, thereby mitigating batch effects across platforms.

### Handling EV heterogeneity

A leading challenge faced by the research community of EVs, which significantly hinders application of ML methods, is the inherent heterogeneity among EV populations [278]. Unlike synthetic NPs, EVs possess great biogenetic heterogeneity with wide ranges of sizes, cargo contents, membrane protein expressions, and cellular origins. This heterogeneity not only indicates their multifaceted physiological roles but also imposes great challenges in terms of model robustness, classification accuracy, and biological interpretability in computational modeling [279, 280].

#### Multifaceted nature of EV heterogeneity

EVs encompass multiple subtypes, including:

- exosomes (~ 30–150 nm), originating from the endosomal pathway [281];
- microvesicles (~ 100–1,000 nm), formed by direct budding from plasma membranes [282]; and.
- apoptotic bodies (> 1000 nm), released during programmed cell death [283].

Within these categories, vesicles derived from a single cell type can vary in molecular composition, dependent on context dependent signals like hypoxia, inflammation, or oncogenic stress. Cargo selection, including proteins, miRNAs, long noncoding (lnc)RNAs, lipids, and metabolites, is carried out with a certain degree of selectivity and is also further regulated by intracellular signaling networks as well as extracellular cues [284].

In addition, EVs from different tissues can have similar sizes and surface antigens, namely CD9 and CD63. This similarity makes it difficult to separate functionally distinct populations using conventional methods, including NTAs or immunoaffinity capture [285].

#### Implications for ML: the curse of biological variability

Structural and molecular heterogeneity causes high label noise and feature overlap in ML workflows. For supervised learning, training data that do not properly reflect the breadth of heterogeneity data can lead to [286]:

- overfitting to dominant EV subtypes while failing to generalize to rarer, potentially more clinically relevant populations;
- misclassification which occurs when subpopulations with unique biological functions have similar biophysical properties; and.
- loss of interpretability, since such models can be based on spurious correlations rather than true mechanistic patterns.

In the case of unsupervised learning methods, such as clustering and dimensionality reduction, high intraclass variance can obscure biologically relevant groupings, leading to poor differentiation of functionally distinct EV subsets.

#### Potential strategies to address heterogeneity in ML EV studies

Researchers are increasingly adopting new methodologies to mitigate the effects of EV heterogeneity in ML models.

- Stratified Sampling and Hierarchical Modeling: Datasets can be structured to include established sources of variability, such as EV size, cellular origin, or disease condition. Hierarchical models, like mixed effects RFs, can handle sample level variations, while highlighting features with generalizability [287].
- Organization by Grouping or Subcategorization: Clustering algorithms, such as K means, density-based clustering of applications with noise (DBSCAN), and Gaussian Mixture Models (GMMs), can be applied before the classification step to identify and distinguish subpopulations of EVs. This is done to enable the training of models on more homogeneous subsets, thus improving performance and biological resolution [288].
- Multi modal Integration: Combining orthogonal data modalities such as imaging, flow cytometry, and proteomics can compensate for limitations of any single feature space. Multi view learning and ensemble strategies can aggregate diverse inputs to create more stable and generalizable models [289].
- Domain Adaptation and Transfer Learning: Techniques such as adversarial training and fine tuning of pretrained models on different datasets can enable batch effect mitigation and improved robustness to new biological environments where EV populations show significant divergence from training data [290].
- Features and Biological Limitations’ Significance. Including known biological knowledge [291], e.g., known sorting motifs or organ specific markers of EVs, can help restrict model behavior and reduce over reliance on features that are confounded or non reproducible [292]. Furthermore, explainability tools, e.g., SHAP, can offer additional insights into factors driving model predictions.

EV heterogeneity exists in reality and cannot be avoided; however, it can be managed, quantified, and utilized. Combining well planned experimental designs and advanced ML methods with a basic understanding of biology is essential for developing EV-based models that can be explained, reproduced, and applied to other areas [293]. Addressing this challenge is not only important for improving the quality of ML systems but is also key for enhancing current knowledge of EVs on a single vesicle level [294].

### Interpretability of ML models

With ML playing a progressively central role in producing insights in the field of EV research, with its applications ranging from diagnostics to the development of therapies, concerns have arisen about the interpretability of these models, especially in the context of clinical decision making [44]. While advanced ML models, including DNNs and ensemble methods, have exhibited remarkable predictive performance, they often behave as “black boxes,” making it difficult to comprehend the reason behind a particular prediction [295]. In high stakes clinical settings, where reproducibility, accountability, and trust are essential, it is necessary that models prove to be transparent and explainable to satisfy regulatory requirements and gain approval from healthcare professionals [296].

#### The necessity of interpretability of EV ML applications

EV biomarkers exhibit considerable complexity, often involving multidimensional omics data, biophysical properties, and descriptors from imaging modalities. However, clinicians require interpretable results that explain relationships between input parameters (e.g., miRNA expressions, particle sizes, and surface markers) and clinical outcomes (e.g., cancer type and responses to therapy) [297]. The utility of predictive models in the clinic is reduced when models fail to provide understandable explanations for their predictions, regardless of their statistical soundness.

In regulatory frameworks, the requirement for explainability is becoming both a legal and ethical imperative. The US Food and Drug Administration [298] and the European Medicines Agency [299] have stated that diagnostic algorithms must provide understandable decision making, particularly when used in risk stratification or in treatment recommendations [300]. Additionally, expectation is growing among patients for open integration of AI within healthcare settings, especially as evidence based tests are nearing routine use in specialties like oncology, neurology, and infectious disease [301, 302].

#### The tradeoff between model complexity and interpretability

There is a basic tradeoff between a model’s complexity and its interpretability. Linear models like LR and LASSO, and decision trees are easy to interpret; however, they might not be the best performing models on high dimensional, non linear EV datasets. DL models (e.g., CNNs for TEM image segmentation or AEs for omics data) excel at capturing complex patterns but lack inherent interpretability [303–305].

#### Model selection should thus be dictated by the application context

a) During an exploratory study or the discovery of a biomarker, a complex model can uncover relationships that might be hidden provided that situations are reversed and the findings are validated [307].

b) For clinical use, it may be better to choose intrinsically interpretable models or hybrid systems containing a black box prediction and clear rationale layers [308].

#### Emerging solutions for explainable AI (XAI) in EV research

Now that EV-based biomarkers and therapies are expected to enter the clinic, this is where the importance of explainability truly becomes non-negotiable. No black box model [308], regardless of its accuracy, is going to be acceptable to clinicians or regulatory officials, or even to patients, unless there is a clear pathway from the characteristics of the EVs to the outcomes. XAI aids this in that each attribution is human-readable, illustrating what particular variables-lipid, protein, miRNA, size, microfluidic data contributed to the estimate of risk, or a medication recommendation, or a quality control failure [309].

Post-hoc techniques such as SHapley Additive exPlanations (SHAP) or Local Interpretable Model agnostic Explanations (LIME) can be directly applied to EV omics and other tabular datasets, where a range of factors must be balanced. These techniques provide a feature attribution for both predictions and groups of patients, identifying the sets of miRNAs, protein markers, or size distributions that drive the classification of respondents vs. non-respondents or benign vs. malignant diseases [310]. When applied to image-based EV assays, attention or saliency techniques (gradient-based saliency, Grad-CAM heatmaps, or transformers) can pinpoint the corresponding spatial patterns, intensities, or sets of channels that drive a given prediction [311]. All these techniques to improve XAI can be combined effectively within the EV platform, including data-driven optimization of the protocol, to increase its medical credibility by enabling domain experts to scrutinize biological feasibility and to provide documentation of medical reasoning [312].

To close this gap, several backward and forward conversion techniques have been developed and implemented in EV related ML pipelines.

- SHAP, also known as SHapley Additive exPlanations, is based on cooperative game theory, which computes an importance value for each feature with respect to a particular prediction [313]. SHAP can be used to identify miRNAs and environmental factors that are closely associated with disease progression, which can provide an excellent prediction model in EV analysis [314].
- LIME: LIME is associated with local variables and manageable interpretations of surrogates, which are simplified in a particular data source [315]. It helps to confirm a prognosis on the basis of LIME such as breast cancer or the symptoms that are severe or invasive [316].
- Attention Mechanisms: Attention mechanisms in ML (e.g. for transcriptomic or sequence based EV sorting motifs) specify which parts of input data are to be focused on, increasing the ability to interpret the biological processes without sacrificing specificity [317].
- The Importance of Features in Tree Based Methods: ML algorithms like RFs and XGBoost inherently produce scores of feature importances that can be mapped and examined for determining important variables between groups, for example, separating cargo proteins or miRNAs between stages of disease [318, 319].
- Surrogates and Model Simplification: Where transparent and effective models are not available, surrogate models operationalized as explainable approximations based on outputs of incomprehensible black box models can be created to provide clinical understanding without sacrificing predictive effectiveness [320].

#### Integration into medical records

Interpretability tools are increasingly being incorporated into EV-based diagnostic platforms. One such example is the EVMAP system, which includes visualization modules that highlight which markers contribute most to a prostate cancer risk score, thus enabling decision making by clinicians [114]. Similarly, some research studies now include interpretability heatmaps or ranked feature lists alongside AUC statistics, acknowledging that the availability of explanations is just as important as the raw performance statistics in translational environments [321].

In EV ML, interpretability is indispensable because it is a prerequisite for reliable, reproducible, and ultimately clinically usefulness of AI. Future work should balance the complexity of algorithms with human interpretability, so that complex biological markers discovered by ML are both statistically robust and clinically actionable.

### Integration of multi omic data

The complex biological properties of EVs stem from their varied molecular compositions, which comprise proteins, RNAs, lipids, metabolites, and DNA that mirror the physiological and pathological states of their donor cells. However, successfully harnessing this complexity requires more than just performing individual omics analyses [322]. To obtain meaningful insights at a systems level, it is imperative to use integrative multi omics strategies that combine these disparate molecular modalities to construct holistic, mechanistic, and predictive models related to the biology and function of EVs. Integrating these, however, presents significant computational, statistical, and algorithmic challenges, especially when integrated into ML platforms [323].

#### The promise of multi omic integration in EV biology

Each omics layer captures a unique aspect of EV function:

- proteomics reveal signaling and surface markers that mediate EV uptake, tropism, and function [324];
- transcriptomics (especially small RNA Seq) captures regulatory miRNAs and mRNA transcripts relevant to intercellular communication [324]; and.
- lipidomics reflect vesicle biogenesis, membrane fluidity, fusion capacity, and disease specific signatures [324].

When taken alone, these types of data can give incomplete views. But when they are combined, they reveal functional cargo modules, regulatory networks, and disease specific EV phenotypes with more certainty. For example, proteomic and miRNA data can show post transcriptional regulatory interactions within EVs, while lipidomic datasets can highlight the trafficking, fusion, or release pathways under different stress conditions (e.g., hypoxia and inflammation) [325, 326].

#### Computational and statistical challenges

Despite their potential, multi omics EV studies are relatively uncommon due to the inherent complexity in data integration. Challenges include the following aspects.

- **Data heterogeneity**: The omics layers defer much in the feature dimension, sparsity, dynamic range, and technical noise. For instance, RNA Seq data may have many features while lipidomic data may have hundreds [327, 328].
- **Sample imbalance and missingness**: Frequently, not all omics categories are observable for each specimen owing to expenses or mechanical issues, which complicate the matrix’s alignment and model completion [329].
- **Non linearity and cross modality interactions**: Relationships between omics layers are usually non linear and depend on the context, and therefore they require flexible modeling frameworks [330, 331].
- **High computational demands**: The study of multimodal modalities often requires a lot of data to be generalizable [325], but most EV studies have small cohorts with a sample size of fewer than 200, increasing the risk of overfitting.

#### ML frameworks for multi omics integration

Several ML paradigms have been adapted to address these integration challenges.

- **Early Integration (Feature level fusion):** Merging omics traits into a a single joined vector to present to an ML model (e.g. an RF or SVM) is very intuitive, but has its own issues, such as feature dominance and scaling bias [332, 333].
- **Late Integration (Decision level fusion)**: Independent models are on each omics type, and their outputs (e.g., probabilities and scores) are via voting, stacking, or LR. This may miss cross omics interactions [334].
- **Intermediate Integration (Latent space models)**: Strategies such as canonical correlation analyses (CCAs), matrix factorization, or AEs translate each omic layer into a shared latent space where subsequent modeling can take place. For instance, a VAE was trained on eight publicly available multi omics datasets [335, 336].
- **Graph Based Integration**: Omics layers are described as multilayered networks, and each of them captures molecular interactions and topological features. Graph neural networks (GNNs) can capture complex relationships between modalities and are considered capable of studying EV driven intercellular communication [337].
- **Kernel Based and Ensemble Approaches**: These models (e.g., Multiple Kernel Learning and multi view boosting) treat each omics layer as a “view” and combine them to improve predictions while preserving modality specific contributions [338].

#### Real world applications in EV research

A new study that combined miRNA, protein, and lipidomic features from EVs of lung adenocarcinoma patients used RF ensembles, trained on singular and combined features, to improve early cancer detection. The combined model achieved an AUC value of 0.96, outperforming all single omics models and identified new cross omics biomarker sets (e.g., miR 92a and multiple ceramide species) which had synergistic diagnostic importance [339, 340].

Mounting evidence suggests the possibility of multi omics signatures from EV-derived multi omics to serve as an early stage sensitive biomarker of AD [341]. Most notably, tau protein pieces within EVs are an indicator of neurofibrillary disease and are measurable in peripheral fluids, or as a noninvasive read on central nervous system degeneration. Among the transcriptomic burden, miR 125b is upregulated in AD and is directly involved in tau hyperphosphorylation and synaptic dysfunction [342], while miR 34a controls neurotoxicity by targeting beta secretase 1 (BACE1), a key amyloid beta metabolism enzyme [343]. Complementing these RNA signatures, dysregulation of phospholipids of ceramide and phosphatidylserine species is involved in amyloid deposition and neuronal apoptosis, further implicating EV lipidomics in disease pathology [344, 345]. Current multi-occurs research can manage to effectively integrate these layers of molecules with supervised ML, i.e., SVM ensembles, for early AD classification. This approach is consistent with the conceptual shift towards EV-based composite biomarkers that capture the intersecting molecular cascades of AD and has promise for clinically acceptable, non-invasive diagnostics [332].

Integration of proteomics, genomics, and lipidomics into EV research poses tremendous potential to decode highly complex biological activities and construct comprehensive disease models [337]. However, to do that, it is essential that sophisticated ML models be introduced that are capable of processing various types of noise, small datasets, and diverse cross-omic effects. Now, as computational power and the size of training datasets improve, multi omics ML can become the bridge between traditional molecular biology and novel clinical practices.

## Future directions

### Development of new ML methods

Unique characteristics of EV datasets are their high dimensionality, heterogeneity, and small sample sizes that pose extreme methodological challenges [346] to which traditional ML techniques are not optimally suited. Consequently, the development of next generation ML models with dedicated designs tailored to EV data have emerged as a principal area of innovation [347]. Such novel algorithms hold potential to extract signals more efficiently, reduce overfitting, and be biologically interpretable while handling cases of sparse, noisy, and multimodal data [348].

#### Challenges specific to EV datasets

EV-based datasets have certain characteristics that differ from common assumptions of ML.

- **High dimensionality with low sample size (HDLSS)**: Omics level features (e.g., miRNA and proteomics) often number in the thousands, yet clinical EV studies typically have < 100–200 samples leading to model overfitting and instability [349].
- **Noise and batch effects**: Technical changes owing to EV separation strategies, reagent sets, and instruments can overshadow true biological effects [350].
- **Heterogeneity in class distributions**: Many datasets have a class imbalance problem and pose significant difficulties in training and evaluating classifiers [351].

In order to resolve these problems, the discipline is moving quickly to make its own DL designs, probabilistic frameworks, and transfer learning paradigms that correctly fit the form and constraints of EV data.

#### Architectures designed for low Number, high dimensional (LNHD) settings

**Autoencoder (AE) Variants with Regularization**: Deep AEs, especially when using sparse and denoising AEs, can be adapted to compress EV omics data into low dimensional latent spaces, which enables robust feature learning despite small sample sizes [351]. Generalization can be achieved by imposing regularization penalties (e.g., L1/L2 norms) or employing variational AEs (VAEs) for sample clustering or anomaly detection [352].

**Few Shot Learning and Meta Learning**: The ability of few shot learning to generate generalizable class prototypes is now being investigated in the context of EV classification with a small amount of labeled data, inspired by recent developments in rare disease modeling [353].

**Bayesian DL for Uncertainty Quantification**: Probabilistic models like Monte Carlo dropout or Bayesian neural networks (BNNs) provide predictions and also confidence intervals that are indispensable for decision making in clinical EV applications. They help differentiate between real signals and model uncertainty, an important ability in the noisy and limited conditions that one may face [354].

**Self-Supervised Learning (SSL)**: SSL methods, like contrastive learning, help in learning useful representations from unlabeled EV data by forming pseudo labels or solving auxiliary tasks (e.g. recreating input features). For imaging applications, simple frameworks for contrastive learning of visual representations (SimCLR) style pipelines have been employed with electron microscopy (EM) of EVs to learn useful representations of EM that can outperform conventional CNNs in situations in which labeled images are scarce [355].

**Graph Neural Networks (GNNs) for Molecular Interactions**: GNNs may offer hope in simulating the relational formation of EV cargos, such as miRNA protein networks or lipid protein co packaging [356]. These simulations focus on EV datasets in the form of graphs rather than plain tables, which helps elucidate interrelations and dependencies of the cargo.

Looking ahead, the convergence of GNN-based network modeling, federated multi-center learning, and real-time EV analytics is likely to define the next generation of ML-enabled EV platforms, evolving fragmented, single-center experiments into privacy-preserving, continuously learning systems that are tightly coupled to clinical workflows.

#### Toward task specific model design

Recent advances in EV ML are now utilizing architecture specific modeling, unlike typical usage from computer vision or natural language processing. A custom implementation called ScanEV was developed by combining the Radon transform with a residual CNN architecture, which is specifically used for segmenting EVs on TEM images, and is more efficient than simple CNNs and human segmentation annotations [357].

Moreover, for flow cytometry based EV classification, combined pipelines that incorporate feature engineering (e.g., t distributed stochastic neighbor embedding (t SNE) for dimension reduction) and boosted decision trees (e.g., XGBoost) were proven to outperform traditional gating based methods, particularly in noisy, low volume datasets [358, 359].

#### Synthetic data and generative modeling

In order to address the issue of data scarcity, generative adversarial networks (GANs) and variational data synthesis are two techniques that are currently being used to create realistic omics or image data for data augmentation [360]. Synthetic samples can be beneficial in improving the robustness of a classifier or simulating rare EV phenotypes for diagnostic modeling [361, 362].

The future of EV research will depend on the data at one’s disposal as well as the ML algorithm used to analyze the data. In particular, custom built ML models will be essential for high dimension, low sample EV analysis. These algorithms can provide insights into EV biology and also meet the requirements for diagnostic and therapeutic use [363].

### AI for personalized medicine

The interface of AI and EV biology is revolutionizing the landscape of personalized medicine. EVs are nanoscale vehicles mediating intercellular communication, packing a great diversity of nucleic acids, proteins, lipids, and metabolites that reflect the real time physiological and pathological state of their source cells. As opposed to fixed genomic information, EV profiles are dynamic in nature, yielding temporal information regarding the progression of disease, responses to therapy, and mechanisms of drug resistance [278]. When measured using AI powered approaches, such vesicle derived biosignatures appear to be strong tools for the purposes of personalized diagnoses, prognostic evaluations, and therapeutic strategic designs.

#### EVs as dynamic biomarkers for precision health

EVs are secreted by nearly all cell types and are found in diverse biofluids, such as plasma, urine [364], saliva [365], and cerebrospinal fluid [366], making them accessible, non-invasive biosources. Crucially, their cargo composition dynamically evolves with disease states, treatment exposure, and immune modulation. These characteristics enable EVs to function as temporal biomarkers, capturing the phenotypic fluidity of a disease at a systems level [367]. For instance, longitudinal studies on miRNAs linked with EVs demonstrated prognostic markers of recurrence in CRC [368, 369]. In NGDs, neuronal EVs high in tau or synaptic proteins have been linked to cognitive declines and drug resistance in AD [370]. The production and analysis of such data using AI enable mapping of personalized patient pathways, an underlying goal of precision medicine [371]. Table 3 provides a curated overview of current AI enabled EV studies across multiple disease contexts, with a notable focus on oncological applications. The use of supervised and DL models on EV-derived transcriptomics, proteomics, and cytometric data has enabled high accuracy cancer diagnoses (e.g., pancreatic, breast, and colorectal cancers), subtyping, and therapy response predictions. Reinforcement and generative models have recently enabled targeted drug delivery and CRISPR based EV engineering.

#### AI frameworks for personalized diagnoses and risk assessments

Supervised learning algorithms, particularly ensemble classifiers like XGBoost and RFs, and DNNs have been applied to EV omics data analysis for developing individual specific diagnostic signatures. A pioneering study by Zhang et al. [219] created a CRISPR augmented platform for EV miRNA detection, named FEVOR, which, when combined with an ML algorithm, achieved a diagnostic performance of over 97% for CRC using stool derived EVs, outperforming conventional biomarkers like CEA and CA19 9 [372]. In breast cancer, for example, a study identified 18 exosomal miRNAs with differential expression between HER2 positive and TNBC patients. Notably, miR 335, miR 422a, and miR 628 exhibited significant differences, with respective AUC values of 0.737, 0.655, and 0.759. Combining these miRNAs improved discrimination between subtypes, achieving sensitivities of 65%–68% and specificities of 81%–84% [373]. Another study identified a panel of five EV-derived miRNAs (miR 21, miR 106b, miR 181a, miR 484, and miR 1260b) that effectively differentiated breast cancer patients from healthy controls. That panel demonstrated high diagnostic accuracy, with AUC values exceeding 0.9 [374].

In cardiovascular disease, ML models trained on plasma EV proteomic profiles [375] (e.g., apolipoproteins and inflammatory markers [376]) can predict myocardial infarction risk within a certain time limit, potentially outperforming standard lipid panels and enabling preemptive clinical interventions [377].

#### Predictive models for therapy responses

AI is also enabling therapeutic personalization by predicting individual responses to drugs based on EV signatures. For example, in non-small cell lung cancer (NSCLC), patients with high EV levels of miR 21 and epidermal growth factor receptor variant III (EGFRvIII) were found to have a poor response to EGFR inhibitors [378]. A DL model integrating EV transcriptomic and proteomic data accurately predicted resistance patterns, guiding oncologists toward alternative regimens [379, 380]. In multiple sclerosis (MS), an RF classifier using EV contained tetraspanins and central nervous system (CNS) specific proteins can predict steroid responsiveness during relapses [381], potentially reducing trial and error in immunosuppressive therapy selection [382]. Beyond monotherapies, AI can integrate EV data to simulate drug synergies, identify biomarkers of adverse effects, and optimize treatment intervals, advancing the notion of digital twins in medicine computational avatars of patient biology used for in silico treatment testing [383].

#### Real time monitoring and adaptive decision support

AI powered EV analytics offer the unique capability of real time, adaptive treatment monitoring. Temporal modeling techniques, such as recurrent neural networks (RNNs) and time series transformers, are being developed to analyze sequential EV profiles and detect subtle shifts indicative of treatment responses, immune evasion, or emerging resistance [384].

By decoding the rich, temporal language of EV cargos, AI enables a shift from population-based treatment models to truly individualized, responsive healthcare. Predictive modeling of EV data combined with clinical variables and longitudinal sampling holds the key to preemptive diagnoses, therapy optimization, and outcome forecasting. As algorithmic frameworks evolve and regulatory infrastructures mature, EV AI platforms are poised to become central pillars of data driven precision medicine [385–387].

### Emerging trends

As diagnostics and therapeutics based on EVs move into clinical practice, a new wave of interdisciplinary innovation is revolutionizing the field, driven by the convergence of AI, bioengineering, and regulatory science [3]. These new directions represent a concerted move away from proof-of-concept studies alone to an emphasis on translational integrity, scalable manufacturing, and integration into real world settings. Key advances include AI optimized drug delivery systems, robotic production of EVs, and standardization strategies to ensure global reproducibility [388].

#### AI enhanced EV drug delivery: from concept to clinic

EVs have emerged as next generation drug carriers, with several advantages over synthetic NPs: intrinsic biocompatibility, immune evasion, and the ability to traverse biological barriers such as the BBB [389]. However, their therapeutic potential has been constrained by bottlenecks in cargo loading, targeting specificity, and delivery efficiency problems well suited for AI interventions [390].

AI is being used to model EV biodistributions, optimize drug loading processes, and design targeted approaches. For instance, reinforcement learning algorithms have been used to iteratively improve the selection of ligands for surface modifications to optimize tissue specific accumulation in silico before wet laboratory validation [391]. Similarly, DL models trained on multimodal biodistribution data are able to predict localization of engineered EVs in different tissues based on cargo type, surface proteins, and routes of administration, thus accelerating the development of precision delivery systems [392].

An illustrative application is the use of generative design algorithms to engineer EVs carrying CRISPR Cas systems for gene editing [385]. By learning from prior encapsulation and release profiles, AI systems can propose EV configurations with maximized nuclear localization and minimal off target toxicity, enabling safer, more effective genetic therapies.

#### Standardization through data driven design of protocols

Despite growing interest in clinical translation, a major barrier to regulatory approval of EV-based products is the lack of standardized isolation, quantification, and potency assessment protocols. Current methods of ultracentrifugation, SEC, and affinity capture produce variable yields and purity profiles, leading to irreproducibility across labs and patient cohorts [393]. AI helps address this by enabling data driven standardization. Protocol optimization models utilize ML algorithms trained on past data concerning EV isolation, including yield, purity, and recovery rates, to suggest workflows specific to given contexts based on the sample type, planned application, and follow up assays [394].

Automated QC systems, such as EVMAP, apply supervised ML models to detect variations in EV compositions, size distributions, or surface marker expressions, thus providing batch to batch consistency within GMP production processes [395].

Federated learning frameworks are under development to harmonize EV data across global centers without compromising patient privacy by training robust, multi-site ML models while preserving data locality [396]. The approaches utilized for the research of EVs agree with goals outlined by the MISEV and EV TRACK initiatives to promote reproducibility, transparency, and data sharing among scientists in the field of EVs [397].

#### Scalability and automation for clinical grade manufacturing

Scaling up the manufacturing of EVs for clinical use calls for automated, real-time monitoring, and AI powered process control. Bioreactor based systems that are fitted with sensor arrays are being merged with ML algorithms to:

- predict yield estimations based on cell viability, metabolic flux, and culture conditions [398, 399];
- employ time series models (such as long /short term memory (LSTM)) for the task of detecting anomalies to identify changes in release and occurrence patterns or contamination of EVs [400]; and.
- support adaptive control systems that can adjust pH, nutrient flows, or harvest timing to maintain the quality of EVs in real time [401].

Additionally, robotic systems can be trained through the process of reinforcement learning to automate EV purification, labeling, and loading, therefore greatly decreasing human error and boosting reproducibility within a big manufacturing setting [402].

**Table 3:** Applications of ML in EV research for disease Diagnosis, Prognosis, and therapeutic design

Application	Data Type	Machine Learning Technique	Reference
Pancreatic Ductal Adenocarcinoma (PDAC) Diagnosis	Plasma EV miRNAs (e.g., miR 95 3p, miR 26b 5p)	Random forest, SVM	Guo et al., 2021: Identified blood small EV miRNAs as differential diagnosis biomarkers for PDAC [403].
Prostate Cancer Grading	Flow cytometry (EV surface markers)	EVMAP (XGBoost, logistic regression)	Fairey et al., 2023: Developed an EV ML analysis platform (EVMAP) to predict high risk prostate cancer [404].
Colorectal Cancer (CRC) Detection	Stool EV miRNA	Ensemble ML	Zhang et al., 2024: Demonstrated that fecal EV miRNA signatures (FEVOR) could serve as potent noninvasive CRC biomarkers [219].
Breast Cancer Subtyping	EV miRNA (liquid biopsy)	Multivariate logistic regression	Santo et al., 2022: Employed ML assisted FTIR analysis of circulating EVs for breast cancer detection [405].
EV-based Drug Delivery Design	Multi omic cargo (proteins, miRNA, and lipids)	Reinforcement learning, deep neural networks	Greenberg et al., 2023: Proposed AI enabled EV precision drug delivery systems [3].
Non-Small Cell Lung Cancer (NSCLC) Therapy Resistance	EV RNA + protein biomarkers	CNNs + multimodal deep learning	Li et al., 2022: Established an ML assisted dual marker detection method in serum small EVs for NSCLC diagnosis and prognosis [406].
Alzheimer’s Disease Prognosis	EV proteomics (CSF/plasma)	Random forest + feature selection	Mustapic et al., 2017: Demonstrated that neuronal EVs enriched with tau proteins could predict cognitive decline [407].
EV Isolation Method Classification	NTA + flow cytometric metrics	Supervised learning (SVM and RF)	Paproski et al., 2023: Applied ML to predict EV isolation methods with over 90% accuracy using EV profiles [395].
EV-based Drug Delivery (CRISPR)	Engineered EV configuration data	Generative models + AI optimization	Lu et al., 2023: Reviewed CRISPR Cas9 delivery strategies with engineered EVs [408].

AI, artificial intelligence; CNN, convolutional neural network; CRC, colorectal cancer; CSF, cerebrospinal fluid; DNN, deep neural network; EV, extracellular vesicle; EVMAP, Extracellular Vesicle Machine Learning Analysis Platform; FTIR, Fourier transform infrared spectroscopy; ML, machine learning; miRNA, microRNA; NTA, nanoparticle tracking analysis; NSCLC, non small cell lung cancer; PDAC, pancreatic ductal adenocarcinoma; RF, random forest; siRNA, small interfering RNA; SVM, support vector machine; XGBoost, eXtreme Gradient Boosting

#### Comparative analysis of ML applications

Applying ML to the analysis of EV data involves a wide variety of applications, ranging from early disease diagnosis, risk prediction, therapeutic optimization, and QC (Table 3). Each of these applications has unique challenges and opportunities that influence the selection of model architectures, data modality preferences, and the likelihood of clinical deployment. This discussion offers a comprehensive analysis of the relative strengths and weaknesses of prevailing ML approaches used in diagnostic and therapeutic applications involving EVs [3].

#### Diagnostic accuracy versus therapeutic improvement

ML applications in EV diagnostics have largely benefited from relatively well structured, single modality datasets (e.g., miRNA expression and flow cytometry surface markers). These models, especially ensemble classifiers (e.g., RF and XGBoost), have demonstrated high diagnostic accuracy in cancer subtyping, neurological disorders, and infectious disease detection, often outperforming conventional biomarkers in sensitivity, specificity, and early detection [409].

For instance, miRNA signatures from feces based EVs (FEVOR) demonstrated 97.4% accuracy in CRC diagnoses upon being used in combination with ensemble learning algorithms, significantly outperforming the clinical utility of CEA and CA19 9 [219]. Similarly, flow cytometric classifiers of EVs related to prostate cancer (EVMAP) demonstrated a better stratification potential compared to PSA [249].

On the other hand, tasks related to therapeutic optimization, i.e., modeling drug delivery efficacy, predicting treatment resistance, or designing EV carriers, typically involve the use of multimodal and sparse datasets. Such tasks often require the use of more advanced ML strategies, such as DL, reinforcement learning, and generative models. While these methods are adept at uncovering hidden feature interactions (e.g., EV surface proteins and biodistributions), their utility is commonly limited by training data concerns, high variance, and lack of interpretability, which hinder direct clinical translation [3].

Whereas diagnostic models are often biased towards replicability and precision, therapeutic models need to offer biological authenticity, generalizability, and increased feedback based on mechanisms over algorithmic and experimental complexities [410].

#### Data specific technique suitability

The effectiveness of any ML model in EV research is tightly coupled with the layout, size features, and noise properties of the input data. Some ML techniques have proven to be especially suitable for different EV data modalities [411].

Transcriptomics and miRNA Data: These high dimensional, structured datasets have been well served by SVM, LR, and RF classifiers, especially when accompanied by dimensionality reduction or regularization. Feature selection techniques (e.g., LASSO and mutual information) help manage sparsity and reduce overfitting in small sample contexts [412]. NTA and Flow Cytometry Data: Structured tabular data with low dimensionality are appropriate for ensemble methods (e.g., XGBoost) and interpretable model methods (e.g., decision trees) that allow efficient classification and quality evaluation through intrinsic feature importance measures [413]. Imaging Data (e.g., TEM and Cryo TEM): These unstructured datasets require convolutional neural networks (CNNs) or residual networks for segmentation and phenotyping tasks. Techniques like transfer learning and contrastive self supervised learning are increasingly used to compensate for limited labeled image sets [414]. Combinations of Several Omics Approaches: The combination of transcriptomics, proteomics, and lipidomics in experimental settings offers great flexibility in the use of deep AEs, multi view learning, and graph neural networks (GNNs), but at the cost of interpretability and with added computational complexity [415].

#### Other cross cutting limitations

- Sample Size Constraints: In most scenarios, a common restriction is the limitation in cohort numbers, thus weakening the model’s generalizability. Thus, techniques such as data augmentation, synthetic interpolation, and few shot learning are deployed to navigate this drawback [416].
- Interpretability: Black box models (e.g., deep neural networks and reinforcement learners) have a stronger performance in more complex tasks compared to their counterpart white box models, but it is their interpretability that can sometimes work against them. These were discussed earlier in this review (Sect. 5.3). For instance, post hoc tools such as SHAP and LIME can be used to provide only partial knowledge about the working of a model. The inherent interpretable aspect of these models is still not as developed [417].
- Validation and Reproducibility: Various ML EV tools that perform well are still not adequately verified with independent sets of patients or medical conditions [418]. The community needs to implement more stringent procedures related to verification, outside verification, and correct compliance with standards of both MISEV and EV TRACK [419].

## Conclusions

### Summary of key findings

The intersection of AI and EV research is no longer speculative; it is rapidly defining the standards, systems, and strategies required for clinical translation. From smart drug delivery systems and federated data sharing to automated GMP workflows, the future of EV-based medicine is being shaped by ML guided innovations. These emerging trends signal a transition from artisanal science to scalable, regulated, and intelligent EV biotechnologies poised for global health impacts. This review highlights the critical contribution of ML towards the realization of the biomedical potential of EVs in a variety of applications. Through the application of sophisticated algorithms and data driven approaches, investigators have made significant advances in augmenting diagnostic accuracy, functional profiling of EVs, quality control, and therapeutic development. Each ML application in EV research is shaped by the biological objective, data modality, and translational context. While diagnostic models leveraging structured omics data have achieved rapid success, therapeutic applications demand more sophisticated architectures and experimental integration. As the field matures, a key direction will be the development of adaptive, interpretable, and generalizable models that are both biologically grounded and clinically viable.

Of special note, the integration of ML with imaging modalities for EVs (e.g., segmentation through TEM and heterogeneity analysis) and omics sciences (e.g., proteomics, transcriptomics, and lipidomics) has allowed the reliable identification of patterns, biomarker discoveries, and predictive modeling, often outperforming traditional statistical methods.

ML driven EV studies have demonstrated real world utility in detecting cancer subtypes, predicting neurodegenerative progression, stratifying cardiovascular risks, and even designing EV-based CRISPR delivery systems. These advances point toward a maturing field, increasingly capable of bridging molecular complexity with clinical decision making.

### Impacts on biomedical research

The convergence of AI and EV biology represents a key milestone in biomedical research. EVs, once thought to be unimportant byproducts of cellular processes, are now recognized as active players that carry molecular messages with great potential for diagnostics and therapeutics. ML provides the underlying paradigm for deciphering their intricate, heterogeneous, and time varying contents.

These skills present new opportunities for:

- noninvasive diagnostics, through liquid biopsy based EV profiling from blood, urine, or stools.
- real time disease monitoring, via longitudinal modeling of EV biomarker shifts.
- personalized medicine, where AI guided analysis of individual EV signatures can inform treatment decisions, monitor response, and detect resistance early; and.
- precision therapeutics, where engineered EVs optimized via reinforcement learning and deep generative models can deliver payloads with organ or tumor specific accuracy.

Such cross-domain integration will catalyze innovations in cancer immunotherapy, neurodegenerative disease monitoring, infectious disease responses, and gene editing delivery. ML has evolved from being a supporting tool to a critical ingredient in the study of EVs. Moving forward into an era of data driven medicine and insights at the nanometer level in biological systems, the deliberate convergence of AI and EVs offers an enormous potential: to turn complexity into simplicity, variability into specificity, and disorder at the molecular level into clinical certainty.

## Data Availability

No datasets were generated or analyzed during the current study.
